# DeCOOC Deconvoluted Hi‐C Map Characterizes the Chromatin Architecture of Cells in Physiologically Distinctive Tissues

**DOI:** 10.1002/advs.202301058

**Published:** 2023-07-28

**Authors:** Junmei Wang, Lu Lu, Shiqi Zheng, Danyang Wang, Long Jin, Qing Zhang, Mingzhou Li, Zhihua Zhang

**Affiliations:** ^1^ CAS Key Laboratory of Genome Sciences and Information Beijing Institute of Genomics Chinese Academy of Sciences and China National Center for Bioinformation Beijing 100101 China; ^2^ School of Life Science University of Chinese Academy of Sciences Beijing 100049 China; ^3^ Livestock and Poultry Multiomics Key Laboratory of Ministry of Agriculture and Rural Affairs College of Animal Science and Technology Sichuan Agricultural University Chengdu 611130 China; ^4^ Animal Breeding and Genetics Key Laboratory of Sichuan Province Institute of Animal Genetics and Breeding Sichuan Agricultural University Chengdu 611130 China; ^5^ Sars‐Fang Centre & MOE Key Laboratory of Marine Genetics and Breeding College of Marine Life Sciences Ocean University of China Qingdao 266100 China

**Keywords:** computational deconvolution, bulk Hi‐C, deep learning, cell type compositions

## Abstract

Deciphering variations in chromosome conformations based on bulk three‐dimensional (3D) genomic data from heterogenous tissues is a key to understanding cell‐type specific genome architecture and dynamics. Surprisingly, computational deconvolution methods for high‐throughput chromosome conformation capture (Hi‐C) data remain very rare in the literature. Here, a deep convolutional neural network (CNN), deconvolve bulk Hi‐C data (deCOOC) that remarkably outperformed all the state‐of‐the‐art tools in the deconvolution task is developed. Interestingly, it is noticed that the chromatin accessibility or the Hi‐C contact frequency alone is insufficient to explain the power of deCOOC, suggesting the existence of a latent embedded layer of information pertaining to the cell type specific 3D genome architecture. By applying deCOOC to in‐house‐generated bulk Hi‐C data from visceral and subcutaneous adipose tissues, it is found that the characteristic chromatin features of M2 cells in the two anatomical loci are distinctively bound to different physiological functionalities. Taken together, deCOOC is both a reliable Hi‐C data deconvolution method and a powerful tool for functional extraction of 3D genome architecture.

## Graphical abstract

Distinguishing cell type‐specific variation in three‐dimensional genome from heterogeneous bulk samples remains challenging. This study reports a deep convolutional neural network‐based method to obtain cell type proportions by deconvolving bulk Hi‐C data. This approach is a reliable Hi‐C data deconvolution method and a powerful tool for the functional exportation of three‐dimensional genome architecture.

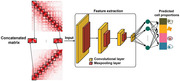

## 1 Introduction

The high‐throughput chromosome conformation capture (Hi‐C)^[^ ^1^ ^]^ technique and its variants have greatly broadened our understanding of 3D genome organization.^[^ ^2^, ^3^, ^4^, ^5^ ^]^ Single‐cell Hi‐C has further advanced our understand of the dynamics of cells in a cell population.^[^ ^6^ ^]^ It has now been widely acknowledged that 3D genome architecture is dynamic and varies substantially from cell to cell.^[^ ^7^ ^]^ Thus, the bulk Hi‐C map of tissue samples may merely represent an average profile over all the complicated cell types it comprises, making it necessary to characterize the 3D genome of each cell type therein. For example, distinguishing the key structural alterations that only occur in cancer cells while seen in the bulk Hi‐C map of a tumor sample may be essential in identifying driver mutations in both cancer research and clinical applications. However, sorting cells from solid tissues into well‐defined cell types remains challenging in most cases,^[^ ^8^ ^]^ leaving bulk sequencing the only applicable experimental option. One way of computationally deriving cell type compositions from bulk Hi‐C data involves deconvolution technologies.^[^ ^9^, ^10^, ^11^ ^]^

To the best of our knowledge, except for Thunder,^[^ ^12^ ^]^ we have yet to find other algorithms published for Hi‐C data deconvolution. Thunder requires a list of predefined marker genes specifically expressed in cell types, that is, cell type‐specific genes, while such gene lists are not always available. Moreover, the accuracy of Thunder failed to compete with that of transcriptome‐oriented methods (see the comparative assessment in this work). A long list of deconvolution algorithms for transcriptome data has already been published in the literature.^[^ ^9^, ^13^ ^]^ Essentially, most transcriptome‐oriented methods assume that the expression levels measured in bulk samples are a mixture, mostly linear, of cell type‐specific expression.^[^ ^9^ ^]^ One pioneering work proposed that the mixture profile may be decomposed by the product of two submatrices, representing cell type‐specific expression profiles and cellular composition, by non‐negative least squares.^[^ ^14^ ^]^ Later, more data decomposition‐based algorithms, such as non‐negative matrix factorization (NMF)‐based methods, ssKL,^[^ ^15^ ^]^ DSA,^[^ ^16^ ^]^ and latent Dirichlet allocation‐based CDSeq,^[^ ^17^ ^]^ also appeared. Deconvolution methods were used to estimate the proportion of each cell type with given bulk and cell type‐specific expression profiles, for example, non‐negative least squares (NNLS),^[^ ^18^, ^19^ ^]^ the robust regression FARDEEP,^[^ ^20^ ^]^ v‐support vector regression (v‐SVR) CIBERSORT (CS),^[^ ^21^ ^]^ DeconRNASeq with quadratic programming,^[^ ^22^ ^]^ and dtangle^[^ ^23^ ^]^ with input data in logarithmic scale. Last, one may also predict the proportion of cell types using cell type‐specific genes, that is, ssKL and DSA.^[^ ^15^, ^16^ ^]^ Our assessment suggested that direct application of these transcriptome‐oriented methods to Hi‐C data may not be reliable (see Section 2); thus, accurately deconvoluting bulk Hi‐C data remains challenging.

Here, we present a convolutional neural network (CNN)‐based algorithm to deconvolve bulk Hi‐C data (deCOOC) to estimate the proportions of cell types in the sample. By comparison with state‐of‐the‐art transcriptome‐oriented tools and Thunder, we demonstrated that deCOOC remarkably outperformed all competitors. Finally, by applying deCOOC, we compared adipose samples between the upper layer of backfat (ULB) and the greater omentum (GOM). Intriguingly, we found that the characteristic chromatin interactions indicated by deCOOC in the two tissues were directly associated with their physiology. Thus, Hi‐C deconvolution could be a powerful tool for the functional exportation of 3D genome architecture.

## 2 Results

### 2.1 A Neural Network‐Based Bulk Hi‐C Matrix Deconvolution Model: deCOOC

**Figure 1:**
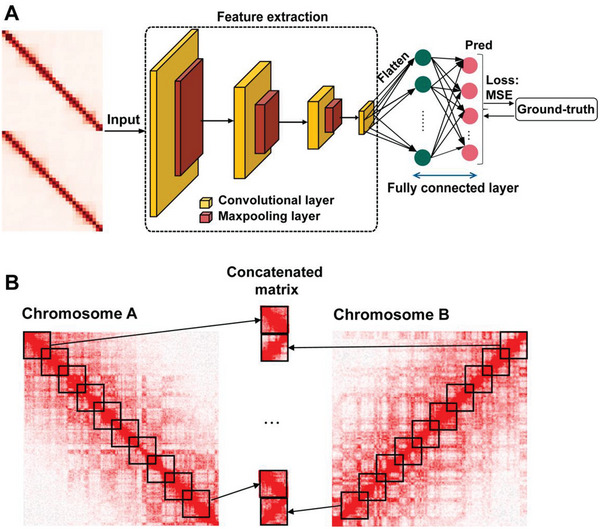
Overview of the deCOOC model. A) Model architecture. The model consists of four convolution layers (each layer of the first three was coupled with one maxpooling layer) and two fully connected neural network layers and the last outputs layer. The input is two square‐like interaction matrices derived from the Hi‐C matrices of two different chromosomes. The last fully connected layer outputs the predicted cell type proportions. Model training and parameter optimization based on Hi‐C data were carried out by minimizing the sum of squares of residues between predicted cell fractions and ground‐truth cell fractions. B) Input design for the model. From the complete Hi‐C matrix (e.g., with a resolution of 500 kb) of one chromosome, multiple square‐like sub‐interaction matrices with fixed sizes (e.g., 30 bins) and steps (e.g., 20 bins) are derived diagonally. Two diagonal sub‐interaction matrices from two chromosomes are stitched together along the row axis.

We developed a CNN‐based model, named deCOOC, to infer the proportions of cell type compositions from bulk Hi‐C data. Briefly, deCOOC consists of four convolution layers, three pooled layers, two fully connected neural network layers and the last output layers (**Figure** **1**A). The deCOOC takes KR (Knight‐Ruiz)^[^ ^24^ ^]^ normalized bulk Hi‐C as input and outputs the final predicted cell composition proportions. Considering the ultrasparse nature of the off‐diagonal region in the Hi‐C matrix[^25^ ^]^ and the variations in chromosome lengths, the input data were formed with only submatrices in the diagonal region, instead of using the full matrix. That is, for a given chromosome, the 30 × 30 submatrices were collected along the diagonal of the Hi‐C matrix with sliding windows (Figure 1B). To avoid plausible variations that might exist between chromosomes, and to mitigate the potential selection of an inappropriate chromosome in the model by introducing a level of hedging (Figure S2, Supporting Information), the final input data for deCOOC were the two concatenated submatrices collected from two different chromosomes. The detailed model description, training process, and parameter selection can be found in the Experimental Section.

To train and test deCOOC, we synthesized training and testing datasets from public single‐cell Hi‐C datasets and in‐house bulk Hi‐C of purified cell line samples. The synthesized data were composed of the mixed cell population with numerous cell composition proportions by randomly sampling from the single cells or purified cell line samples (Figure S1, Supporting Information and Experimental Section). The deCOOC is robust to parameter settings. For example, the size, sliding steps or coordinates of the two matrices picked from the two chromosomes do not substantially affect performance (Figure S3A,B, Supporting Information). In the present work, chromosomes 9 and 11 were randomly taken as examples. However, the choice of chromosome has little effect on performance, as similar results could be seen with other random combinations of chromosomes (Table S1 and Figure S4, Supporting Information).

To assess deCOOC, we compared it with various state‐of‐the‐art deconvolution algorithms. In addition to Thunder, the sole Hi‐C deconvolution tool published,^[^ ^12^ ^]^ we also compared eight transcriptome data‐oriented methods, that is, CS,^[^ ^21^ ^]^ CDSeq,^[^ ^17^ ^]^ DeconRNAseq,^[^ ^22^ ^]^ DSA,^[^ ^16^ ^]^ dtangle,^[^ ^23^ ^]^ FARDEEP,^[^ ^20^ ^]^ NNLS,^[^ ^19^ ^]^ and ssKL.^[^ ^26^ ^]^ Some tools can take bulk Hi‐C data as input directly, for example, CDseq, while others require additional cell type‐specific genes, for example, DSA and ssKL. To adapt these tools to Hi‐C data, we constructed chromatin interaction profiles (CIPs) to mimic the transcriptome data. Differential interaction regions were identified to mimic cell type‐specific gene markers (Figure S6, Supporting Information). The performance of deCOOC was quantified by the root mean square error (RMSE) and a modified version of Lin's concordance correlation coefficient (CCC, see Experimental Section).^[^ ^27^ ^]^ We assessed deCOOC with two single‐cell Hi‐C datasets, that is, mouse cell cycle data (denoted as mCC)^[^ ^7^ ^]^ and human front cortex datasets (denoted as HFC),^[^ ^6^ ^]^ as well as in‐house‐generated bulk Hi‐C data from pig fat tissue cell lines.

### 2.2 The deCOOC Robustly Deconvolutes a Hi‐C Map of Synthesized Bulk Samples with Simple Cell Composition

We assessed deCOOC with synthesized bulk Hi‐C data from the experimental single‐cell Hi‐C dataset mCC, which contains only four cell cycle phases, that is, G1, early‐S, later S‐G2, and M,^[^ ^7^ ^]^ and 11 combinations with more than two cell stages can be fully censused. We synthesized 100 samples for each combination with randomly generated proportions for each cell stage, resulting in 1100 samples. A sample is a cell population with 1000 single cells randomly sampled from mCC, representing a synthesized bulk Hi‐C library. Two‐thirds and one‐third of the 1100 samples were taken as training and testing data, respectively. Performance assessment was presented with the average from fivefold cross‐validation.

**Figure 2:**
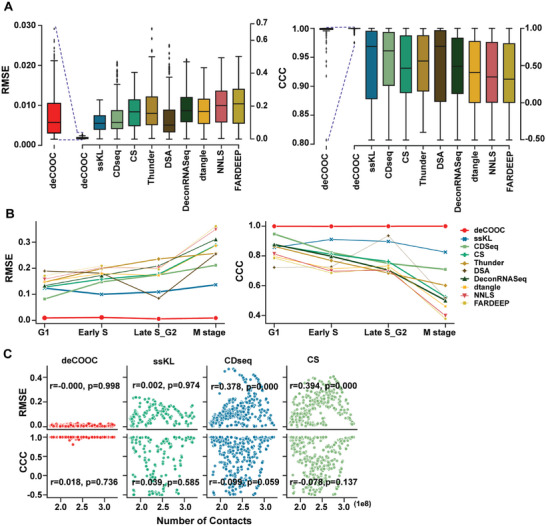
deCOOC performs better (lower RMSE and higher CCC^[^ ^27^ ^]^) on simulated mouse data than other methods. A) Boxplots of RMSE and CCC values over all test bulk samples from deCOOC and other deconvolution algorithms for the simulated mCC test dataset. B) Lineplots of RMSE and CCC values for each cell type. Each symbol represents the RMSE or CCC value between ground‐truth and predicted cell fractions for one cell type. C) Scatterplots of RMSE (CCC in bottom row) values and the number of Hi‐C contacts for simulated mouse data with deCOOC, ssKL, CDSeq, and CS. Pearson correlation coefficients and *p* values are given above the plots. Low RMSE and high CCC values represent good prediction performance of the method. For all algorithms, the number of test samples *n* = 363.

The deCOOC achieved the lowest average RMSE and the highest average CCC, followed by ssKL, CDSeq, and CS (**Figure** **2**A). The predictions by deCOOC were nearly identical to the proportions of ground truth, as it yielded an average RMSE less than 0.008 and CCC larger than 0.990, while the average RMSE and CCC were larger than 0.10 and smaller than 0.54, respectively, for all ssKL, CDSeq, and CS. Similar results were also observed for cell stage‐specific performance (Figure 2B). Although the other tools generated relatively better CCC (≈0.90–0.94) in G1, S, and G2, it remained smaller than 0.83 in the M stage.

Next, we assessed data volume dependency against the performance of tools by calculating Pearson's correlation between accuracy and number of contacts in each dataset for deCOOC, ssKL, CDSeq, and CS (Figure 2C). We found that the CCCs were independent of data resolution for all four tools. For RMSE, the independencies were only seen in deCOOC and ssKL, while weak, but significant, correlations were observed in CS and CDseq (Figure 2C), and such dependencies can also be seen in other tools, for example, Thunder and DSA (Figure S7, Supporting Information). Together, deCOOC is the only tool that predicts cell composition from synthesized bulk Hi‐C from mCC data robustly and accurately.

### 2.3 The deCOOC Robustly Deconvolutes the Hi‐C Map of Synthesized Bulk Samples with Underrepresented Training Data

We assessed the tools with publicly available single‐cell Hi‐C data on the human frontal cortex (HFC), which contains 14 cortical cell types, as an example,^[^ ^6^ ^]^ to test whether deCOOC can infer a heterogeneous cell population. As the number of combinations of all 14 cell types (16354, *n* ≥ 2) is too large to be simulated with limited computational resources, HFC is a perfect dataset to assess the generalization ability of deCOOC. In our experiment, we generated 1300 samples with 892 combinations. After removing 14 samples with only one cell type, we obtained 1286 samples, representing 5.5% of the whole combination space (892 out of 16354, Table S2, Supporting Information, see Experimental Section).

**Figure 3:**
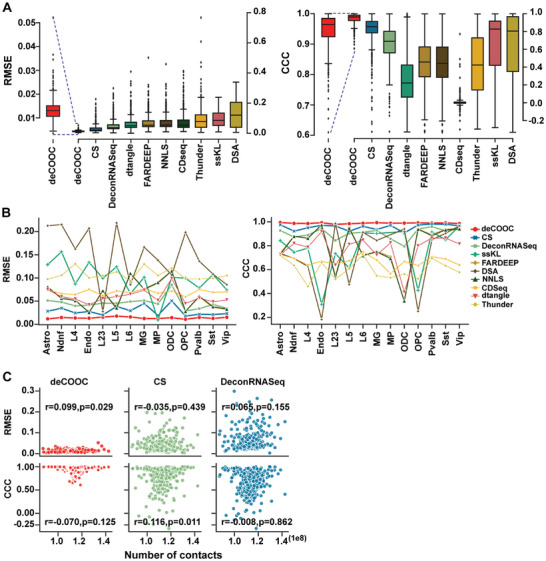
deCOOC behaves more robustly on simulated HFC data than the other methods. A) Boxplots of RMSE and CCC values over all test bulk samples from deCOOC and other deconvolution algorithms for the simulated HFC test dataset. B) Lineplots of RMSE and CCC values for each cell type. Each symbol represents the RMSE or CCC value between ground‐truth and predicted cell fractions for one cell type. C) Scatterplots of RMSE (CCC in bottom row) values and the number of Hi‐C interaction contacts of simulated HFC bulk data with deCOOC, CS, and DeconRNASeq. Pearson correlation coefficients and *p* values are given above the plots. For HFC data, the number of test samples *n* = 486.

The deCOOC performed well in these synthesized heterogeneous cell populations. The deCOOC obtained the highest average CCC and the lowest average RMSE, followed by CS and DeconRNASeq (**Figure** **3**A). Notably, except for deCOOC, the order of performance was substantially different between mCC and HFC for all tools. For example, ssKL and CDSeq were the top two tools with mCC data, while the top two were CS and DeconRNAseq with HFC. Next, we assessed the accuracy of the prediction for each cell type. The deCOOC achieved nearly optimal predictive power for all cell types in terms of CCC and RMSE (Figure 3B), followed, again, by CS and DeconRNAseq. For robustness results, all tested tools showed little dependency on data resolution (Figure 3C and Figure S8, Supporting Information). Given the correlation observed in mCC data, we think that those transcriptome‐based methods could only occasionally be affected by sequencing depth.

Next, we assessed the generalization ability of deCOOC, that is, how much it may work with unseen combinations far from those training samples in the cell type combination space. Underrepresentative sampling may bring local combinatory bias caused by stochastic fluctuation. To obtain such distal samples, we employed Euclidean distance to define the distance between samples. By randomly generating combinations with random cell proportions, we calculated the minimal distances to the existing samples and noticed a periodic distribution (Figure S9B, Supporting Information). Then, the distal unseen samples were defined as the samples with minimal distances larger than the third peak, representing approximately the top 10% of distal newly generated samples (red line in Figure S9B, Supporting Information). We randomly selected 200 samples from the unseen data for further verification. Remarkably, even for those distal unseen samples, deCOOC achieved good performance with a mean RMSE of 0.011 and CCC of 0.993 (Figure S9C, Supporting Information). Notably, deCOOC can quickly converge to the optimal status with such predictive power for unseen samples, as it reached saturated performance with only ≈700–800 training samples (Figure S9D,E, Supporting Information). Taken together, deCOOC is a sensitive network that can be easily generalized to the whole sample space, even with underrepresented training data for heterogeneous cell populations.

### 2.4 Predictive Chromatin Structural Features Utilized by deCOOC Might Be Latent

**Figure 4:**
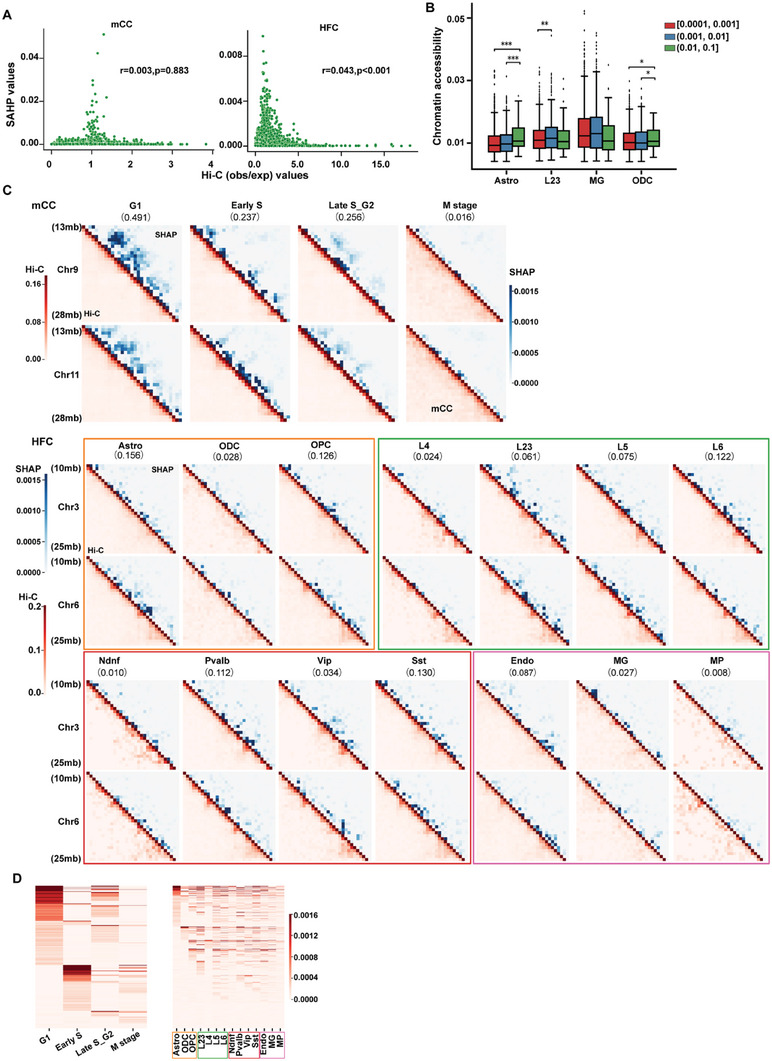
SHAP analysis for model interpretation. A) Scatterplots show weak correlation between SHAP values and Hi‐C (observed/expected) for mCC and HFC examples (e.g., examples are the same as those shown in Figure 4C). Pearson correlation coefficients and *p* values are given above the plots. B) Correlation analysis between chromatin accessibility and SHAP values based on the HFC dataset. Chromatin accessibility was significantly higher in the group with higher SHAP values (i.e., (0.01,  0.1)) than in the other two groups for Astro and ODC cell types. The L23 cell type showed that chromatin openness only in the median SHAP values group (0.001, 0.01) was dramatically greater than that in the lower SHAP values group. The correlation between SHAP values and chromatin accessibility is dependent on different cell types. *P* values were calculated using a one‐sided Wilcoxon signed‐rank test. C) Examples of paired Hi‐C matrix (lower left) and SHAP value maps (upper right) for each cell type of mCC and HFC. The regions of 13–28 mb and 10–25 mb of the two chromosomes for the mCC and HFC bulk examples are shown. Cell types and chromosome numbers are labeled at the top and left of the plots, respectively, while the fraction of each cell type is presented in parentheses. For the HFC example, the fourteen cell types were sorted into four categories (labeled by four rectangles of different colors) according to the clustering of cell‐type specific chromatin interactions.^[^ ^6^ ^]^ D) Heatmap of SHAP values (example shown in C) for each cell type prediction (left for mCC example, right for HFC example). Each row in the heatmap indicates the SHAP values for an interaction site. (Significant differences: *P < 0.05, **P < 0.01, ***P < 0.001).

To assess the contributions of chromatin features to deconvolution, we performed SHapley Additive exPlanations (SHAP) analysis.^[^ ^28^ ^]^ SHAP assigns a number to each feature, representing the importance of the feature in machine learning models,^[^ ^29^, ^30^ ^]^ that is, a positive SHAP value indicates a valid feature in inferring cell types. We calculated SHAP values for all samples in both mCC and HFC datasets and asked what type of chromatin features may be associated with high SHAP values. We presented the results on chromosomes 9 and 11 from mCC and chromosomes 3 and 6 from HFC as examples (**Figure** **4**).

We found that neither normalized Hi‐C contact frequency nor chromatin accessibility significantly contribute to the predictive power of deCOOC. To investigate the potential relationship between chromatin accessibility and the predictive capability of deCOOC, we calculated the correlation coefficient between them. However, the correlation between the Hi‐C map and SHAP values was extremely weak, with an average Pearson correlation coefficient (PCC) of 0.034 and 0.046, and standard deviations of 0.023 and 0.019 for mCC and HPC, respectively (Figure 4A). Specifically, we sought to determine whether the chromatin accessibility of a cell line exhibited a positive correlation with the importance of the features used to predict the proportions of the cell types. To explore this, we compared the SHAP values with bulk ATAC‐seq data in four cell lines (Astro, L23, MG, and ODC) for which publicly available ATAC‐seq data were accessible. Interestingly, the relationship between SHAP values and chromatin accessibility appeared to vary among different cell types. In ODC and Astro, a weak positive association was observed when SHAP values were extremely high, occurring in approximately the top 3–5% range (0.01, 0.1). However, in L23, the opposite pattern was observed, where the association shifted when the SHAP values were low (0.0001, 0.001) compared to (0.001, 0.01) (see Figure 4B). These findings indicate that the chromatin features utilized by deCOOC are not simply contact frequency or chromatin accessibility.

The features that deCOOC utilizes for prediction may, instead, largely be associated with latent functional chromatin structure that distinguishes different cell types. Although Hi‐C map‐based clustering can group the cells into four known categories in the frontal cortex, the borders between cell types within a category remain fuzzy.^[^ ^6^, ^31^ ^]^ However, even with nearly indistinguishable Hi‐C maps within the categories, SHAP profiles are nearly mutually exclusive between them (Figure 4C,D; Figure S10A, Supporting Information), implying a strong association between the latent chromatin structure captured by deCOOC and cell identity. This association might be functional, as evidenced by GO analysis, that is, the genes located in bins with high SHAP values are enriched for GO terms functionally relevant to cells. For example, genes in bins of high SHAP values (top 10%) for G1 were enriched for the regulation of cilium assembly and nitric oxide biosynthetic processes^[^ ^32^, ^33^, ^34^ ^]^ (Figure S10B, Supporting Information). Another example was the regulation of TOR signaling,^[^ ^35^, ^36^ ^]^ and actin cytoskeleton reorganization was found to be enriched in astrocyte‐specific high‐SHAP genes^[^ ^37^ ^]^ (Figure S10C, Supporting Information). Together, instead of relying on cell‐type‐specific chromatin contacts, deCOOC successfully characterized cell type specificity from a seemingly identical Hi‐C map (Figure 4D; Figure S10D, Supporting Information). In addition, we conducted model training using input matrices of various sizes (Figure S11, Supporting Information). Our findings revealed that the bins exhibiting high SHAP values were predominantly shared across different matrix sizes. This observation strongly suggests that the SHAP analysis remained largely unaffected by the size of the input matrix.

### 2.5 Fine‐Tuned deCOOC Deconvolutes Bulk Hi‐C Data of Real Tissue Samples

In most common practical scenarios, single‐cell Hi‐C data are rarely accessible, particularly for solid tissue samples. Therefore, we wondered whether deCOOC could deconvolute bulk Hi‐C without being trained by single‐cell data. By assuming that Hi‐C profiles of a cell type do not substantially differ in situ between the tissue and cultured cell lines, we assessed this performance using pig adipose tissue as a model. Four major cell types appear in most adipose tissues,^[^ ^38^ ^]^ that is, Adi (adipocytes), VEC (vascular endothelial cells), M1 (M1‐type macrophages), and M2 (M2‐type macrophages). We generated high‐quality in situ Hi‐C data for the four cell types of which Adi, M1, and M2 were derived from stem cells and VEC was isolated from the pulmonary aorta (Figure S12 and Table S3, Supporting Information). Similar to what we did with the mCC and HPC datasets, deCOOC was trained and tested with synthesized bulk data from cell lines (Experimental Section). We compared deCOOC with the two best‐performing peer predictors, CDSeq, and CS, according to the assessment we showed above.

**Figure 5:**
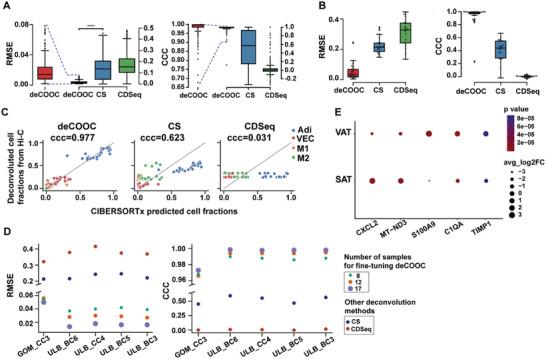
deCOOC performs better than CS and CDseq (lower RMSE, but higher CCC) on pig tissue Hi‐C data. A) Boxplots of RMSE and CCC values from deCOOC, CS, and CDSeq for simulated pig bulk Hi‐C data (randomly sampled experimental Hi‐C contacts of four pig cell lines with artificially produced cell fractions). B) Boxplots of RMSE and CCC for assessing deconvolution performance of the three algorithms on real pig tissues. The deconvolution of CS and CDSeq was conducted on all 22 real adipose samples, and the deconvolution of deCOOC was performed five times on five samples randomly selected from 22 adipose samples (the remaining 17 samples were used to fine‐tune the model), which was performed five times. C) Scatterplots of CIBERSORTx‐predicted cell fractions (*x*‐axis) and deconvoluted cell fractions (*y*‐axis) from fine‐tuned deCOOC, CS, and CDSeq on real samples. The corresponding CCC values for the three methods are presented above the plots. D) RMSE and CCC values of deconvolution on five real test tissues (*x*‐axis) from the three deconvolution methods. deCOOC was fine‐tuned using different numbers of real tissues. E) Differential expression on a log 2 scale of five genes for subcutaneous adipose tissue (SAT) and visceral adipose tissue (VAT). (Significant differences: ****P < 0.0001).

The deCOOC outperformed CS and CDseq, as assessed by RMSE and CCC (**Figure** **5**A). Although the tools were trained by the data generated from cultured cell lines, the performance of all tools has a similar trend and is comparable to that trained by the data from single cells. The RMSE of deCOOC was comparable to that of HPC and significantly larger than that of mCC (Figure 5A). The RMSEs of CS and CDseq were both similar to those with single‐cell datasets (Figure 5A). The CCCs of deCOOC and CS were also comparable to those with both single‐cell datasets, while the CCC of CDseq was nearly zero, on average, similar to that with HPC.

Next, we applied deCOOC to bulk Hi‐C data generated from two pig adipose tissues, that is, the ULB at subcutaneous adipose (SAT) and GOM at abdominal visceral adipose tissue (VAT) , representing physiologically distinguishable subcutaneous and visceral fat, respectively. In total, 12 and 10 samples were from ULB and GOM, respectively, and the corresponding high‐quality in situ Hi‐C and RNA‐seq data were generated (Table S4, Supporting Information). As the deconvolution problem has long been addressed with transcriptome data,^[^ ^9^, ^39^ ^]^ we used CIBERSORTx inferred cell composition as the refs. [40, 41]. By transfer learning, deCOOC, which was pretrained on the above synthesized data, was fine‐tuned with 17 out of 22 adipose samples (12 ULB + 10 GOM) (Experimental Section; Figure S13, Supporting Information). Fine‐tuned deCOOC was then tested on the remaining five samples. Compared to CS and CDSeq, deCOOC had the highest average CCC and lowest RMSE (CCC: 0.937, RMSE: 0.052, Figure 5B). For all cell proportions, deCOOC achieved the highest CCC and lowest RMSE (CCC: 0.975, 95% CI: 0.963–0.983, RMSE: 0.070) (Figure 5C). Furthermore, to further validate our approach, we presented an additional example using the HFC dataset (see in the Supporting Information and Figure S14, Supporting Information).

The number and quality of samples used for fine‐tuning may have had a minor effect on the performance of deCOOC. Generally, the more samples used the better performance one may achieve. For both RMSE and CCC, four out of five testing samples showed better performance when deCOOC was fine‐tuned by more samples (Figure 5D). However, the difference remains minor; for example, the largest differences were 0.026 and 0.012 in RMSE and CCC, respectively, when fine‐tuned by 8 and 17 samples, respectively. One sample (GOM_CC3) was rather stable, irrespective of the number of fine‐tuned samples, which raises the question of whether sample quality may have an effect on the fine‐tuning process. However, when we used different numbers of samples for fine‐tuning, we found that neither CCC nor RMSE was affected to any noticeable degree (Figure S15A, Supporting Information). Nevertheless, for all sample numbers in the random sampling we tested, the CCC and RMSE of deCOOC were larger than 0.980 and less than 0.06, respectively, substantially better values than those of both CS and CDSeq (Figure 5D; Figure S15A, Supporting Information). This result suggests that deCOOC is suitable for performing the deconvolution task on real bulk Hi‐C of heterogeneous tissues.

### 2.6 deCOOC Deconvoluted the Chromatin Architecture in M2 Cells, Reflecting Inflammation‐ and Energy Metabolism‐Related Functionality of Visceral and Subcutaneous Fat, Respectively

Finally, we asked if the chromatin feature that deCOOC utilizes to define a cell type is functionally relevant. Because visceral fat is more inflammation‐related than subcutaneous fat,^[^ ^42^ ^]^ we used M2 cells in GOM tissue as an example to examine this question. M2 macrophages are anti‐inflammatory macrophages that promote the resolution of inflammation, coordinate tissue integrity, and release anti‐inflammatory mediators;^[^ ^43^, ^44^ ^]^ importantly, they are specifically active and enriched in visceral fat. Interestingly, within the positive SHAP regions for M2 cells in GOM, we found more inflammation‐related GO terms significantly enriched for genes in GOM than in ULB (Figure S15B, Supporting Information). More evidence came from gene expression analysis in which inflammatory factor genes, for example, S100A9, C1QA, and TIMP1, showed higher expression in visceral adipose tissue (VAT) than in subcutaneous adipose tissue (SAT) (Figure 5E; Figure S15F, Supporting Information).^[^ ^45^, ^46^, ^47^ ^]^

On the other hand, it is known that subcutaneous fat is more involved in energy metabolism than visceral fat. We found that energy‐related metabolism GO terms (i.e., mitochondrion organization and positive regulation of angiogenesis) were significantly enriched with genes in the positive SHAP regions for M2 cells in the ULB (Figure S15B, Supporting Information). Similarly, energy metabolism‐related genes, for example, MT‐ND3 (crucial for mitochondrial function,^[^ ^48^ ^]^ and CXCL2 (regulation of angiogenesis),^[^ ^49^ ^]^ were found to be more highly expressed in SAT than in VAT (Figure 5E; Figure S15F, Supporting Information). Together, these examples suggest that deCOOC is not only a powerful tool for deconvoluting bulk Hi‐C into cell type components, but also capable of detecting potential functional relevance in chromatin features.

## 3 Discussion

In this study, we proposed deCOOC, a CNN‐based cellular deconvolution model, to map Hi‐C of tissues to cellular compositions. Our assessment demonstrated that deCOOC outperformed existing methods in prediction accuracy and robustness.

To the best of our knowledge, Thunder is the only published method on Hi‐C deconvolution.^[^ ^12^ ^]^ As an unsupervised model, Thunder does not involve a training process, which allows it to perform Hi‐C deconvolution with a predefined number of cell types. This is critical in cases where the cellular composition is complicated or unknown. However, this advantage pays the price with relatively low predictive accuracy, that is, even when the number of cell types is given, Thunder can only achieve performance similar to that of transcriptome‐oriented methods (Figure 2).

We employed CNN instead of canonical feature selection‐based methods to perform Hi‐C deconvolution based on the following rationale. First, Hi‐C, among cell types, is known to be largely conserved,^[^ ^50^ ^]^ and the cell type‐specific features are not always easily detectable and sensitive to sequencing depth. Second, CNN‐based methods are known to be capable of capturing latent characters from data. These latent characteristics are important not only for prediction, but they also hint at functionality that may lead to novel discovery. Finally, the CNN‐based method is more robust to experimental variations (Figure 2).

Several aspects of deCOOC need to be improved in the future. First, cultured or single‐cell Hi‐C data from purified cells remain essential in deCOOC. However, in real practice, neither of these two types of training data may be easily accessible. Furthermore, fine‐ tuning is required as deCOOC was trained completely by simulated data, which does not couple with unavoidable and unpredictable experimental variations. That is why we employed transfer learning technology in fine‐tuning our pig adipose data. However, this fine‐tuning may be sensitive to sample and data quality. Second, the bin size we used in the present work was 500 kb. This bin size prevented us from examining the contributions of high‐resolution chromatin structures, for example, TAD/Loop, to deconvolution. However, at this resolution, we demonstrated that deCOOC was rather robust to data volume. Moreover, we tried to associate the chromatin compartment with SHAP values with negative results (data not shown), which may imply that it might be the features beyond simple chromatin structures that contribute towards deconvolution.

The 3D chromatin structure has been considered a promising direction for the functional interpretation of noncoding variants that contribute to human traits and disease etiology.^[^ ^51^, ^52^, ^53^, ^54^ ^]^ However, the highly dynamic nature of chromatin loops between cell types puts another dimension of complexity into this canonical genotype‐phenotype association problem.^[^ ^55^, ^56^ ^]^ That is, to associate a noncoding variant with its target through spatial approximation of chromatin, it is essential to refer to the chromatin structure in the target cell type instead of bulk data, which are commonly available in most conditions. With the increased categories of cell types and cell states interrogated by the scHi‐C approach,^[^ ^57^ ^]^ we anticipate that future integrative analysis of bulk data with matched scHi‐C data of heterogeneous samples will generate biologically informative estimates of cell‐type‐ and states‐specific interactome and help interpret findings of genome‐wide association studies (i.e., variant‐to‐function) by assigning a previously unrecognized function to the noncoding variants at single‐cell resolution in population scales. However, deCOOC serves as a starting point to address the problem with its power to deconvolute bulk Hi‐C of tissue samples with heterogeneous cell components. Potential future directions, including Hi‐C profile predictions and differential chromatin structure identifications between samples, should be in the range of the canon for computational genomics in the field.

## 4 Experimental Section

### Animals

The seven‐day‐old Bama minipig used in this study was obtained from the experimental farm of Sichuan Agricultural University for primary cell isolation and culture. Pigs were treated humanely and sacrificed by anesthesia until death, and the bone marrow and pulmonary aorta were separated and used for cell culture. The animal experiment was approved by the Experimental Animal Ethics Committee of Sichuan Agricultural University (Approval No. 20200176) and performed following the guidelines for the management and use of laboratory animals.

### Cell Culture—Isolation and Culture of Macrophages

Two phenotype macrophages, M1 and M2, were differentiated from bone marrow stem cells (BMSCs).^[^ ^58^, ^59^, ^60^ ^]^ Briefly, bone marrow cells were obtained by puncture and passed through a 40 µm cell strainer (FALCON, USA, 352340). After removing erythrocytes with an ACK lysate kit (Thermo Fisher Scientific, USA, A1049201), bone marrow cells were seeded and cultured with DMEM/F12 (Thermo Fisher Scientific, USA, 11330‐0320) supplied with 10% heat‐inactivated fetal bovine serum (FBS) (Thermo Fisher Scientific, USA, 10099141C), 100 U mL^−1^ penicillin, and 100 mg mL^−1^ streptomycin (Thermo Fisher Scientific, USA, 030311B) (DMEM/F12 10% FBS) at 37 °C and 5% CO_2_. After 4 h, the unattached cells were enriched and inoculated in a new flask. M1 and M2 macrophage differentiation was performed in a new flask by induction with 20 ng mL^−1^ GM‐CSF (Absin, China, abs04132) (M1 macrophages) or 20 ng mL^−1^ M‐CSF (Absin, China, abs00846) (M2 macrophages), respectively.^[^ ^58^, ^59^, ^60^ ^]^

### Cell Culture—Isolation and Culture of Adipocytes

Primary adipocytes were obtained following a bone marrow stem cell (BMSC) differentiation protocol using the OriCell Human Umbilical Cord Blood Mesenchymal Stem Cell Adipose Differentiation Induction Medium Kit (Cyagen, USA, HUXUB‐90031) as previously described.^[^ ^61^ ^]^

### Cell Culture—Isolation and Culture of Vascular Endothelial Cells (VECs)

In this study, VECs were isolated from the pulmonary aorta as previously described.^[^ ^62^ ^]^ Briefly, the two ends of blood vessels were ligated with sutures and digested with 0.1% type I collagenase at 37 °C for 20 min. After digestion, collected VECs were seeded in flasks and cultured with DMEM (Thermo Fisher Scientific, USA, 11330‐0320) supplemented with 10% heat‐inactivated fetal bovine serum (FBS) (Thermo Fisher Scientific, USA, 10099141C), 100 U mL^−1^ penicillin and 100 mg mL^−1^ streptomycin (Thermo Fisher Scientific, USA, 030311B) (DMEM 10% FBS) at 5% CO_2_ and 37 °C.^[^ ^62^ ^]^

### In Situ Hi‐C Library Preparation

Hi‐C libraries for the indicated cells were generated according to a previously published protocol with some minor modifications.^[^ ^50^ ^]^ The obtained cells were incubated with 4% formaldehyde at room temperature (20–25 °C) for 30 min for chromatin crosslinking, and then glycine was added to obtain a final concentration of 0.25 mol L^−1^ to quench the formaldehyde. The mixture was then centrifuged at 3000 × *g* for 5 min at 4 °C and suspended in 100 µL of cold 1 × PBS. Approximately 100 000 cells were pipetted into a new tube for subsequent experiments, spun for 5 min at 3000 × *g* at 4 °C. Nuclei of formaldehyde‐fixed cells were permeabilized, and DNA was digested with ten units of MboI (a 4‐cutter restriction enzyme) for 5 h at 37 °C. The restriction fragment overhangs were filled, labeled by biotinylated nucleotides, and then ligated in a small volume. After cross‐link reversal, the ligated DNA was purified and sheared to a length of 300–500 bp at which point ligation junctions were pulled down with streptavidin beads and prepped for Illumina NovaSeq 6000 sequencing.

### Single‐Cell Hi‐C(scHi‐C) Datasets

Two scHi‐C datasets were used with different features: mouse embryonic stem cell (mESC) cells (Nagano et al., 2017^[^ ^7^ ^]^) and HFC brain prefrontal cortex cells (Lee et al., 2019^[^ ^6^ ^]^) (see Supporting Information for more details).

### Preprocessing and Quality Control for Sequencing Reads of Pure Cell Lines

The quality of all libraries was assessed using FastQC (http://www.bioinformatics.babraham.ac.uk/projects/fastqc/). Raw fastq files were first processed in paired‐end mode with trim_galore (v. 0.6.6). For Hi‐C libraries, the first 15 base pairs (bp) were trimmed from the 5′‐end of both read 1 and read 2 owing to their low complexity. Reads with a mean quality score less than, or equal to, 30 were removed, and fragments with lengths shorter than 30 bp were discarded. Then, the trimmed reads were converted into “.hic” files by Juicer tools.^[^ ^63^ ^]^

Cell Culture—Reproducibility Analysis for These Four Cell Lines

GenomeDisco^[^ ^64^ ^]^ scores between replicates were greater than 0.83 among all chromosomes for all cell types with a bin size ≥ 100 kb. A resolution of 500 kb was used in this study.

### In Silico Generation of Extra scHi‐C Data

When there were sufficient single‐cell Hi‐C data available for certain cell types, a straightforward approach of randomly sampling cells from the existing dataset was employed. However, a challenge of imbalanced cell type proportions in cases where certain cell types dominated some bulk samples, while having limited real single‐cell data available was faced. To address this challenge and ensure a more balanced representation of cell types in the simulated bulk samples, a strategy to generate simulated single‐cell Hi‐C data for specific cell types using the existing scHi‐C data was devised. This strategy aimed to enhance the diversity and complexity of the scHi‐C data, thereby improving the overall diversity of the simulated bulk samples.

For the mCC and HFC datasets, Hi‐C contacts were aggregated from all single cells belonging to the same cell type to construct the bulk Hi‐C profiles for each cell type. To determine the number of simulated scHi‐C contacts for a given cell type, the median number of scHi‐C contacts across all single cells of that cell type was defined. Subsequently, in silico scHi‐C contacts were generated by downsampling the predetermined number of contacts from the pooled bulk Hi‐C data (Figure S16, Supporting Information).

### Simulation of Bulk Hi‐C Samples

To generate simulated bulk Hi‐C samples for training, two subsets of samples were created. One subset contained all cell types, while the other subset included only a subset of cell types (e.g., randomly selecting two or three out of the four cell types).

### Generation of Cell Type Compositions

The fractions of each cell type in the simulated samples were determined using the random.rand() function from the Python NumPy package. This approach allows for the generation of random proportions without prior knowledge of cellular compositions within tissues.

For each cell type (i), a random number (*r_i_*) was chosen from a uniform distribution between 0 and 1. The random fraction of the cell type (*f_i_*, rounded to three decimal places) was then calculated as the following

(1) $$f_{i}\;=\;\frac{r_{i}}{\sum_{1}^{K}r_{i}}$$

where *K* represents the number of cell types contained in the sample.

### Determining the Number of Single Cells for Simulated Bulk Samples

For the mCC and HFC datasets, a total of *N*_sum_ =  1000 single cells were composed in silico to mimic a bulk Hi‐C sample. Hence, the number of cells required for sampling from the scHi‐C dataset for each cell type *i* can be obtained using the equation

(2) $$N_{i}=\;\left[\;N_{\mathrm{sum}}\times \;f_{i}\right]$$

### Generating Simulated Bulk Samples for Bama Miniature Pig Data

Due to the varying number of sequencing contacts in each bulk Hi‐C sample, the number of contacts for each simulated bulk sample was sampled from a uniform distribution within a specified numerical range. Specifically, the range was set between 148 054 505 (minimum) and 235 282 624 (maximum), based on the actual number of sequencing contacts in pig tissues.

Let *M*_sum_ denote the total number of contacts in a simulated bulk sample. Then, the number of contacts required to be sampled from the bulk contacts of the pure cell line for cell type *i* could be calculated using the equation

(3) $$M_{i}\;=\;\left[\;M_{\mathrm{sum}}\;\times \;f_{i}\;\right]$$

All sampled contacts from each cell line were merged together to form the contacts of a simulated bulk sample. These contacts were then converted into the “.hic” format, which is compatible with Juicer tools.

### Generation of Reference Matrices for the Deconvolution

Given the Hi‐C interactions of any two cell types, HiCcompare^[^ ^65^ ^]^ (v1.8.0) was employed to derive differential chromatin interactions (e.g., chromatin region1 region2: chr1 500000 55000000; adjusted p value <0.05) (Figure S3, Supporting Information). These differential interactions were integrated from all chromosomes for related cell types as chromatin interaction profiles (CIPs). For example, for the bulk sample mixed with three cell types (A, B, and C), the differential chromatin interactions between A & B, A & C and B & C were integrated to make the CIP.

### Algorithm Comparison

The root mean square error (RMSE), Lin's CCC,^[^ ^27^ ^]^ and Pearson correlation coefficient *r* between real and predicted cell fractions were measured. These metrics are defined by

(4) $$\mathrm{RMSE}\;\left(y,\;\hat{y}\right)=\;\sqrt{\sum_{i\;=\;1}^{n}\frac{{\left({\hat{y}}_{i}-y_{i}\right)}^{2}}{n}}$$

(5) $$\mathrm{CCC}\left(y,\;\hat{y}\right)\;=\;\frac{2\mathrm{cov}\left(y,\;\hat{y}\right)\;}{\sigma_{y}^{2}+\sigma_{\hat{y}}^{2}+{\left(\mu_{y}\;-\;\mu_{\hat{y}}\right)}^{2}}$$

where ***y*** is the vector of ground‐truth fractions, $\hat{y}$ is the vector of predicted fractions, *n* is the number of elements in each vector, σ_***y***_ is the standard deviation (SD) of ***y***, $cov(y,\;\hat{y})\;$is the covariance of ***y*** and $\hat{y}$, and μ_***y***_ and $\mu_{\hat{y}}$ are the means of the ground‐truth and predicted fractions, respectively. This calculated version of CCC was used for evaluating the predicting concordance of cell types.

As the sum value of cell type proportions for each bulk Hi‐C sample equals one, μ_***y***_ and $\mu_{\hat{y}}$ will always be equal (e.g., $\frac{1}{n}$), which could increase the CCC values for each bulk sample. Therefore, CCC was slightly modified by replacing ${\left(\mu_{y}\;-\;\mu_{\hat{y}}\right)}^{2}$ with the mean square error between the predicted fractions and ground truth (shown below) to assess the predicting concordance correlation for each Hi‐C bulk sample

(6) $$\mathrm{Modified}\;\mathrm{CCC}\left(y,\;\hat{y}\right)\;=\;\frac{2\mathrm{cov}\left(y,\;\hat{y}\right)\;}{\sigma_{y}^{2}+\sigma_{\hat{y}}^{2}+\sum_{i\;=\;1}^{n}\frac{{\left({\hat{y}}_{i}-y_{i}\right)}^{2}}{n}}$$

### deCOOC Workflow

Step 1: Data preprocessing: First the KR normalized Hi‐C matrix was scaled into a probabilistic matrix‐like form, in which each row and column shall sum to one. Let *V_ij_* denote the (*i*, *j*) element in the matrix; it can be scaled as

(7) $$V_{ij\_\mathrm{scaled}}=\;\frac{V_{ij}}{S_{i}}$$

where *S_i_* denotes the sum of row *i*.

The input matrices for deCOOC are rectangular matrices concatenated from two square submatrices by rows. The two submatrices were taken from the diagonal of the Hi‐C contact matrices of two different chromosomes (Figure 1B). Unless otherwise stated, the submatrices have size = 30 bins (Figure 1B).

Step 2: deCOOC model building: As shown in Figure 1A, deCOOC has four convolutional layers and two dense neural network layers. Except for the output layer, tanh was used as the nonlinear activation function. TensorFlow (v2.4)^[^ ^66^ ^]^ and Keras (v2.4) (https://keras.rstudio.com/articles/why_use_keras.html) of Python (v3.7) were used to implement deCOOC.

Step 3: Training and prediction: The model was trained by fivefold cross‐validation. Parameters were optimized using Adam^[^ ^67^ ^]^ with a learning rate of 0.001. Mean square error (MSE) was chosen as the loss function and RMSE as the metric during the training stage. The optimization objective was to minimize MSE. During the training process, a technique named ReduceLROnPlateau from the Keras.callbacks package that monitored RMSE and optimized the learning rate (the parameter monitor was set as “val_root_mean_squared_error”, patience was set as four, and the factor was set as 0.98) was also employed. Early stopping regularization was used to prevent overfitting (Figure S5, Supporting Information).

### Fine‐Tuning for the Pig Dataset

The model was fine‐tuned based on transfer learning.^[^ ^68^ ^]^ deCOOC was pretrained using simulated pig bulk Hi‐C data. During fine‐tuning, all layers, except fully connected dense layers in the trained model, were frozen so that their weights could not be updated. Then, the network was retrained on a small number of real pig samples using a very small learning rate (0.0004), so the parameters of the fully connected layers could be updated. Five‐fold cross‐validation was adopted in the model development stage, which produced five sets of parameters for deCOOC on simulated pig bulk samples, five models with different initializations (five sets of parameters) were fine‐tuned using real samples (Figure S13, Supporting Information). Predicted results on real pig samples were averaged by these five fine‐tuned models.

### SHAP‐Based Interpretability Analysis

SHAP (SHapley Additive exPlanations)^[^ ^28^ ^]^ encompasses a collection of techniques employed for elucidating the output of deep learning and machine learning models. To identify the key features influencing each specific input pertaining to the various possible outputs (cell type compositions) for deCOOC, the GradientExplainer method was utilized.^[^ ^28^ ^]^ This approach enabled to ascertain the most significant features associated with each input.

During the model training process, a 5×1‐fold cross‐validation technique was employed to determine SHAP values. This methodology involved creating five separate models during the training phase, where each model was trained on a unique subset of the data. To evaluate the significance of features for each potential output, the GradientExplainer method was utilized.

For each acquired model, the GradientExplainer was applied to both the model itself and the training data specific to that model. This step allowed to generate a GradientExplainer object for each model, facilitating the subsequent computation of SHAP values.

Next, the respective GradientExplainer objects were utilized to calculate SHAP values on the testing data. This entire process was repeated for all five models, resulting in five distinct sets of SHAP values. To provide a comprehensive interpretation of the SHAP values, the average of these five sets were then computed, yielding the final SHAP values for further analysis and interpretation.

### Correlation of SHAP Values and Values in the Hi‐C Matrix

To show the direct relationship between SHAP values and Hi‐C, the Pearson correlation between the normalized (observed/expected) Hi‐C and SHAP values by the function “scipy.stats.pearsonr()” was calculated. Twenty bulk samples were randomly selected for the mCC and HFC datasets. Bulk Hi‐C of each cell line was generated according to their composition in the bulk samples. For each sample, the Pearson correlation between Hi‐C (obs/exp) and positive SHAP values were calculated for all cell types present in the sample. In addition, correlation coefficients for 20 samples were averaged for mCC and HFC separately.

### Analysis of SHAP Values Based on ATAC‐seq

For HFC, ATAC‐seq data^[^ ^69^ ^]^ of four cell lines (Table S7) from UCSC was obtained. The open fraction (*O*_f_) of each bin was determined by

(8) $$O_{f}=\;\frac{\sum L_{r}}{500\;\mathrm{kb}}$$

where *L*_r_ indicates the length of open chromatin regions (at the bin), and 500 kb represents the total length of the bin. Bins with *O*_f_ less than 0.008 were filtered. The open fraction for each element off the diagonal in the SHAP values matrix was defined by

(9) $$O_{f_{i,j}}=\;\frac{O_{f_{i}}+O_{f_{j}}}{2}$$

where *i* and *j* represent the row and column numbers of the element located in the SHAP matrix, and $O_{f_{i}}$ denotes the open fraction of the *i*th bin in the SHAP values matrix.

### GO Enrichment Analysis

RefSeq genes for mm9 and hg19 (for mCC and HFC datasets, respectively) were obtained from UCSC Table Browser, and genes were defined by the region including 2.5 kb upstream and downstream of the TSS (transcription start site) region. Bedtools^[^ ^70^ ^]^ were used to obtain the list of genes of interest for the genomic region of interest through SHAP value analysis. Then, Metascape was used and GO biological processes in the Pathway option were chosen for enrichment analysis.

### Single‐Cell RNA‐seq Data Analysis

Processed data files were downloaded from Gene Expression Omnibus (GEO) with accession number GSE129363.^[^ ^71^ ^]^ Seurat v.4.2.0 was then used for downstream analysis of filtering, normalization, clustering, dimensionality reduction, and differential gene expression. Cells with low gene (<200), low unique molecular identifier (UMI, <200), and high mitochondrial gene expression (>20%) were removed. To exclude doublets, cells with high gene (>2500) and high UMI (>13750) were also removed. In addition, only nondiabetic samples were included for further analysis in this article, which contained 14 370 cells. The top 20 dimensions were used to generate the final clusters using principal component analysis (PCA) and graph‐based clustering (Figure S15C, Supporting Information). Genes expressed in a minimum of 20% of cells in either of the test populations were considered for analysis. Differential gene expression analysis between cell populations was performed using the Wilcoxon rank sum test in the “FindMarkers” function. The top 100 genes in each cluster were identified in comparison with all other cells using the function “FindAllMarkers” in Seurat, while keeping a cutoff of p_adj ≤ 0.01. According to this paper,^[^ ^71^ ^]^ immune clusters (1, 4, 7, 8, 12, and 15) were identified using overlapping the expressed markers of each cluster (Figure S15C, Supporting Information) with the immune markers it provided. The immune cell populations consisted of 5000 (34.7%) cells. Clustering was performed again for the immune cells, producing subclusters for SAT and VAT (shown as Figure S15D, Supporting Information). Cluster 6 was defined as the M2 cell type, which was marked by high expression of FOLR2 and KLF4^[^ ^71^, ^72^, ^73^ ^]^ (Figure S15E, Supporting Information).

### Statistical Analysis

To test the correlation between the algorithms’ performance and the number of contacts of samples, the Pearson correlation coefficient was derived by the function scipy.stats.pearsonr(). The one‐sided Wilcoxon signed‐rank test was used to compare metrics (i.e., RMSE and CCC) of different algorithms evaluated on exactly the same data, in which *p* values were calculated by the scipy.stats.wilcoxon() function in Python. Statistical significance calculations for model's input were performed using a two‐sided Wilcoxon signed‐rank test (scipy.stats.wilcoxon() function in Python). For analyses combining SHAP values and ATAC‐seq data, Mann‒Whitney U one‐sided tests were conducted using the scipy.stats.mannwhitneyu() function in Python. (Significant differences: *P < 0.05, **P < 0.01, ***P < 0.001, ****P < 0.0001).

## Conflict of Interest

The authors declare no conflict of interest.

## Author Contributions

J.W. and L.L. are joint first authors. J.W., M.L., and Z.Z. conceived this project. J.W. developed deCOOC. J.W., Q.Z., and J.L. analyzed the data. L.L. and S.Z. performed the experiments. J.W., L.L., Q.Z., M.L., and Z.Z. prepared the manuscript. All authors read and approved the final manuscript.

## Supporting information

Supporting Information

Supplemental Table 1

Supplemental Table 2

## Data Availability

The data that support the findings of this study are openly available in National Genomics Data Center (NGDC) at https://ngdc.cncb.ac.cn/gsa/s/8s786ujg, reference number 9039.

## References

[1] M. Gates , U. S. Agency , G. Miiro , J. Serwanga , A. Pozniak , D. Mcphee , W. Jaoko , J. Dehovitz , L. G. Bekker , P. Pitisuttithum , J. Virol. 2009, 83, 7337.19439467

[2] V. Y. Goel , A. S. Hansen , Wiley Interdiscip. Rev.: Dev. Biol. 2021, 10, e395. 32987449 10.1002/wdev.395 PMC8236208

[3] G. Ron , Y. Globerson , D. Moran , T. Kaplan , Nat. Commun. 2017, 8, 2237. 29269730 10.1038/s41467-017-02386-3 PMC5740158

[4] J. Dekker , L. A. Mirny , Cell 2016, 164, 1110. 26967279 10.1016/j.cell.2016.02.007 PMC4788811

[5] H. Zheng , Biol. Mood Anxiety Disord. 2019, 20, 535.

[6] D. S. Lee , C. Luo , J. Zhou , S. Chandran , A. Rivkin , A. Bartlett , J. R. Nery , C. Fitzpatrick , C. O'Connor , J. R. Dixon , J. R. Ecker , Nat. Methods 2019, 16, 999. 31501549 10.1038/s41592-019-0547-z PMC6765423

[7] T. Nagano , Y. Lubling , C. Varnai , C. Dudley , W. Leung , Y. Baran , N. Mendelson Cohen , S. Wingett , P. Fraser , A. Tanay , Nature 2017, 547, 61. 28682332 10.1038/nature23001 PMC5567812

[8] Y. Wang , N. E. Navin , Mol. Cell 2015, 58, 598. 26000845 10.1016/j.molcel.2015.05.005 PMC4441954

[9] F. Avila Cobos , J. Vandesompele , P. Mestdagh , K. De Preter , Bioinformatics 2018, 34, 1969. 29351586 10.1093/bioinformatics/bty019

[10] A. E. Teschendorff , C. E. Breeze , S. C. Zheng , BMC Bioinf. 2017, 18, 105. 10.1186/s12859-017-1511-5 PMC5307731 28193155

[11] A. J. Titus , R. M. Gallimore , L. A. Salas , B. C. Christensen , Hum. Mol. Genet. 2017, 26, R216. 28977446 10.1093/hmg/ddx275 PMC5886462

[12] B. Rowland , R. Huh , Z. Hou , C. Crowley , J. Wen , Y. Shen , M. Hu , P. Giusti‐Rodriguez , P. F. Sullivan , Y. Li , PLoS Genet. 2022, 18, e1010102. 35259165 10.1371/journal.pgen.1010102 PMC8932604

[13] S. Mohammadi , N. Zuckerman , A. Goldsmith , A. Grama , Proc. IEEE 2017, 105, 340.

[14] D. Venet , F. Pecasse , C. Maenhaut , H. Bersini , Bioinformatics 2001, 17, S279. 11473019 10.1093/bioinformatics/17.suppl_1.s279

[15] R. Gaujoux , C. Seoighe , Infect., Genet. Evol. 2012, 12, 913. 21930246 10.1016/j.meegid.2011.08.014

[16] Y. Zhong , Y. W. Wan , K. Pang , L. M. Chow , Z. Liu , BMC Bioinf. 2013, 14, 89. 10.1186/1471-2105-14-89 PMC3626856 23497278

[17] K. Kang , Q. Meng , I. Shats , D. M. Umbach , M. Li , Y. Li , X. Li , L. Li , PLoS Comput. Biol. 2019, 15, e1007510. 31790389 10.1371/journal.pcbi.1007510 PMC6907860

[18] C. L. Lawson , R. J. Hanson , S*olving Least Squares Problems* , Prentice‐Hall, Englewood Cliffs, NJ 1974.

[19] C. L. Lawson , R. J. Hanson , Solving Least Squares Problems, Society for Industrial and Applied Mathematics, Philadelphia 1995.

[20] Y. N. Hao , M. Yan , B. R. Heath , Y. L. Lei , Y. Y. Xie , PLoS Comput. Biol. 2019, 15, e1006976. 31059559 10.1371/journal.pcbi.1006976 PMC6522071

[21] A. M. Newman , C. L. Liu , M. R. Green , A. J. Gentles , W. Feng , Y. Xu , C. D. Hoang , M. Diehn , A. A. Alizadeh , Nat. Methods 2015, 12, 453. 25822800 10.1038/nmeth.3337 PMC4739640

[22] T. Gong , J. D. Szustakowski , Bioinformatics 2013, 29, 1083. 23428642 10.1093/bioinformatics/btt090

[23] G. J. Hunt , S. Freytag , M. Bahlo , J. A. Gagnon‐Bartsch , Bioinformatics 2019, 35, 2093. 30407492 10.1093/bioinformatics/bty926

[24] P. A. Knight , D. R. Ima , J. Numer. Anal. 2013, 33, 1029.

[25] B. R. Lajoie , J. Dekker , N. Kaplan , Methods 2015, 72, 65. 25448293 10.1016/j.ymeth.2014.10.031 PMC4347522

[26] J. P. Brunet , P. Tamayo , T. R. Golub , J. P. Mesirov , Proc. Natl. Acad. Sci. U. S. A. 2004, 101, 4164. 15016911 10.1073/pnas.0308531101 PMC384712

[27] L. I. Lin , Biometrics 1989, 45, 255. 2720055

[28] S. M. Lundberg , S. I. Lee , NeurIPS Proc. 2017, 30, 4765.

[29] J. X. Mi , A. D. Li , L. F. Zhou , IEEE Access 2020, 8, 191969.

[30] A. Adadi , M. Berrada , IEEE Access 2018, 6, 52138.

[31] T. E. Bakken , N. L. Jorstad , Q. Hu , B. B. Lake , W. Tian , B. E. Kalmbach , M. Crow , R. D. Hodge , F. M. Krienen , S. A. Sorensen , J. Eggermont , Z. Yao , B. D. Aevermann , A. I. Aldridge , A. Bartlett , D. Bertagnolli , T. Casper , R. G. Castanon , K. Crichton , T. L. Daigle , R. Dalley , N. Dee , N. Dembrow , D. Diep , S. L. Ding , W. Dong , R. Fang , S. Fischer , M. Goldman , J. Goldy , et al., Nature 2021, 598, 111. 34616062 10.1038/s41586-021-03465-8 PMC8494640

[32] O. V. Plotnikova , E. N. Pugacheva , E. A. Golemis , Methods Cell. Biol. 2009, 94, 137. 20362089 10.1016/S0091-679X(08)94007-3 PMC2852269

[33] C. Napoli , G. Paolisso , A. Casamassimi , M. Al‐Omran , M. Barbieri , L. Sommese , T. Infante , L. J. Ignarro , J. Am. Coll. Cardiol. 2013, 62, 89. 23665095 10.1016/j.jacc.2013.03.070

[34] M. J. Ford , P. L. Yeyati , G. R. Mali , M. A. Keighren , S. H. Waddell , H. K. Mjoseng , A. T. Douglas , E. A. Hall , A. Sakaue‐Sawano , A. Miyawaki , R. R. Meehan , L. Boulter , I. J. Jackson , P. Mill , R. L. Mort , Dev. Cell 2018, 47, 509. 30458140 10.1016/j.devcel.2018.10.027 PMC6251972

[35] J. Wahis , M. G. Holt , Front. Cell. Neurosci. 2021, 15, 645691. 33716677 10.3389/fncel.2021.645691 PMC7947346

[36] M. G. Garelick , B. K. Kennedy , Exp. Gerontol. 2011, 46, 155. 20849946 10.1016/j.exger.2010.08.030 PMC3432286

[37] J. Schiweck , B. J. Eickholt , K. Murk , Front. Cell. Neurosci. 2018, 12, 261. 30186118 10.3389/fncel.2018.00261 PMC6111612

[38] L. Jin , Q. Tang , S. Hu , Z. Chen , X. Zhou , B. Zeng , Y. Wang , M. He , Y. Li , L. Gui , L. Shen , K. Long , J. Ma , X. Wang , Z. Chen , Y. Jiang , G. Tang , L. Zhu , F. Liu , B. Zhang , Z. Huang , G. Li , D. Li , V. N. Gladyshev , J. Yin , Y. Gu , X. Li , M. Li , Nat. Commun. 2021, 12, 3715. 34140474 10.1038/s41467-021-23560-8 PMC8211698

[39] F. Avila Cobos , J. Alquicira‐Hernandez , J. E. Powell , P. Mestdagh , K. De Preter , Nat. Commun. 2020, 11, 5650. 33159064 10.1038/s41467-020-19015-1 PMC7648640

[40] A. M. Newman , C. B. Steen , C. L. Liu , A. J. Gentles , A. A. Chaudhuri , F. Scherer , M. S. Khodadoust , M. S. Esfahani , B. A. Luca , D. Steiner , M. Diehn , A. A. Alizadeh , Nat. Biotechnol. 2019, 37, 773. 31061481 10.1038/s41587-019-0114-2 PMC6610714

[41] C. A. Glastonbury , A. C. Alves , J. S. E. Moustafa , K. S. Small , Am. J. Hum. Genet. 2019, 104, 1013. 31130283 10.1016/j.ajhg.2019.03.025 PMC6556877

[42] A. El‐Wakkad , M. H. Nel , H. Sibaii , S. R. El‐Zayat , Cytokine 2013, 61, 682. 23306429 10.1016/j.cyto.2012.11.010

[43] T. Roszer , Mediators Inflammation 2015, 2015, 816460. 10.1155/2015/816460 PMC4452191 26089604

[44] A. Viola , F. Munari , R. Sanchez‐Rodriguez , T. Scolaro , A. Castegna , Front. Immunol. 2019, 10, 1462. 31333642 10.3389/fimmu.2019.01462 PMC6618143

[45] Y. Liu , L. Zou , H. Tang , J. Li , H. Liu , X. Jiang , B. Jiang , Z. Dong , W. Fu , Front. Cardiovasc. Med. 2022, 9, 791875. 35433892 10.3389/fcvm.2022.791875 PMC9008490

[46] L. H. Chen , J. F. Liu , Yan‐Lu, X. Y. H.e , Chi‐Zhang, H. H. Z. , Front. Oncol. 2021, 11, 642144. 34079754 10.3389/fonc.2021.642144 PMC8166322

[47] T. Vogl , M. Eisenblätter , T. Völler , S. Zenker , S. Hermann , P. van Lent , A. Faust , C. Geyer , B. Petersen , K. Roebrock , M. Schäfers , C. Bremer , J. Roth , Nat. Commun. 2014, 5, 4593. 25098555 10.1038/ncomms5593 PMC4143994

[48] D. Voet , J. G. Voet , C. W. Pratt , Fundamentals of Biochemistry : Life at the Molecular Level, Wiley, Hoboken, NJ 2013.

[49] C. L. Addison , D. A. Arenberg , S. B. Morris , Y. Y. Xue , M. D. Burdick , M. S. Mulligan , M. D. Iannettoni , R. M. Strieter , Hum. Gene Ther. 2000, 11, 247. 10680839 10.1089/10430340050015996

[50] S. S. P. Rao , M. H. Huntley , N. C. Durand , E. K. Stamenova , I. D. Bochkov , J. T. Robinson , A. L. Sanborn , I. Machol , A. D. Omer , E. S. Lander , E. L. Aiden , Cell 2014, 159, 1665. 25497547 10.1016/j.cell.2014.11.021 PMC5635824

[51] J. S. Baxter , O. C. Leavy , N. H. Dryden , S. Maguire , N. Johnson , V. Fedele , N. Simigdala , L.‐A. Martin , S. Andrews , S. W. Wingett , I. Assiotis , K. Fenwick , R. Chauhan , A. G. Rust , N. Orr , F. Dudbridge , S. Haider , O. Fletcher , Nat. Commun. 2018, 9, 1028. 29531215 10.1038/s41467-018-03411-9 PMC5847529

[52] A. Chesi , Y. Wagley , M. E. Johnson , E. Manduchi , C. Su , S. Lu , M. E. Leonard , K. M. Hodge , J. A. Pippin , K. D. Hankenson , A. D. Wells , S. F. A. Grant , Nat. Commun. 2019, 10, 1260. 30890710 10.1038/s41467-019-09302-x PMC6425012

[53] S. Nurk , S. Koren , A. Rhie , M. Rautiainen , A. V. Bzikadze , A. Mikheenko , M. R. Vollger , N. Altemose , L. Uralsky , A. Gershman , S. Aganezov , S. J. Hoyt , M. Diekhans , G. A. Logsdon , M. Alonge , S. E. Antonarakis , M. Borchers , G. G. Bouffard , S. Y. Brooks , G. V. Caldas , N. C. Chen , H. Cheng , C. S. Chin , W. Chow , L. G. de Lima , P. C. Dishuck , R. Durbin , T. Dvorkina , I. T. Fiddes , G. Formenti , et al., Science 2022, 376, 44.35357919

[54] S. Aganezov , S. M. Yan , D. C. Soto , M. Kirsche , S. Zarate , P. Avdeyev , D. J. Taylor , K. Shafin , A. Shumate , C. Xiao , J. Wagner , J. McDaniel , N. D. Olson , M. E. G. Sauria , M. R. Vollger , A. Rhie , M. Meredith , S. Martin , J. Lee , S. Koren , J. A. Rosenfeld , B. Paten , R. Layer , C. S. Chin , F. J. Sedlazeck , N. F. Hansen , D. E. Miller , A. M. Phillippy , K. H. Miga , R. C. McCoy , et al., Science 2022, 376, eabl3533. 35357935 10.1126/science.abl3533 PMC9336181

[55] A. S. Hansen , C. Cattoglio , X. Darzacq , R. Tjian , Nucleus 2018, 9, 20. 29077530 10.1080/19491034.2017.1389365 PMC5990973

[56] M. Gabriele , H. B. Brandao , S. Grosse‐Holz , A. Jha , G. M. Dailey , C. Cattoglio , T. S. Hsieh , L. Mirny , C. Zechner , A. S. Hansen , Science 2022, 376, 496. 35420890 10.1126/science.abn6583 PMC9069445

[57] T. Zhou , R. Zhang , J. Ma , Annu. Rev. Biomed. Data Sci. 2021, 4, 21. 34465168 10.1146/annurev-biodatasci-020121-084709

[58] P. Bjorck , Blood 2001, 98, 3520. 11739152 10.1182/blood.v98.13.3520

[59] M. Leidi , E. Gotti , L. Bologna , E. Miranda , M. Rimoldi , A. Sica , M. Roncalli , G. A. Palumbo , M. Introna , J. Golay , J. Immunol. 2009, 182, 4415. 19299742 10.4049/jimmunol.0713732

[60] T. McLaughlin , S. E. Ackerman , L. Shen , E. Engleman , J. Clin. Invest. 2017, 127, 5. 28045397 10.1172/JCI88876 PMC5199693

[61] G. P. Freitas , A. T. P. Souza , H. B. Lopes , R. L. B. Trevisan , F. S. Oliveira , R. R. Fernandes , F. U. Ferreira , F. A. Ros , M. M. Beloti , A. L. Rosa , Bio‐Protoc. 2020, 10, e3534. 33654758 10.21769/BioProtoc.3534 PMC7842647

[62] Y. L. Zhao , C. J. Zhao , D. K. Coopers , Y. Lu , K. W. Luo , H. Y. Wang , P. F. Chen , C. C. Zeng , S. D. Luan , L. S. Mou , H. C. Gao , J. Visualized Exp. 2019, 150, e59673. 10.3791/59673 31475978

[63] N. C. Durand , M. S. Shamim , I. Machol , S. S. Rao , M. H. Huntley , E. S. Lander , E. L. Aiden , Cell Syst. 2016, 3, 95. 27467249 10.1016/j.cels.2016.07.002 PMC5846465

[64] O. Ursu , N. Boley , M. Taranova , Y. X. R. Wang , G. G. Yardimci , W. S. Noble , A. Kundaje , Bioinformatics 2018, 34, 2701. 29554289 10.1093/bioinformatics/bty164 PMC6084597

[65] J. C. Stansfield , K. G. Cresswell , V. I. Vladimirov , M. G. Dozmorov , BMC Bioinf. 2018, 19, 279. 10.1186/s12859-018-2288-x PMC6069782 30064362

[66] M. Abadi , P. Barham , J. Chen , Z. Chen , A. Davis , J. Dean , M. Devin , S. Ghemawat , G. Irving , M. Isard , M. Kudlur , J. Levenberg , R. Monga , S. Moore , D. G. Murray , B. Steiner , P. Tucker , V. Vasudevan , P. Warden , M. Wicke , Y. Yu , X. Zheng , *arXiv:1605.08695*, 2016.

[67] D. Kingma , J. Ba , arXiv:1412.6980 , 2017.

[68] S. J. Pan , Q. A. Yang , IEEE Trans. Knowl. Data Eng. 2010, 22, 1345.

[69] A. Nott , I. R. Holtman , N. G. Coufal , J. C. M. Schlachetzki , M. Yu , R. Hu , C. Z. Han , M. Pena , J. Xiao , Y. Wu , Z. Keulen , M. P. Pasillas , C. O'Connor , C. K. Nickl , S. T. Schafer , Z. Shen , R. A. Rissman , J. B. Brewer , D. Gosselin , D. D. Gonda , M. L. Levy , M. G. Rosenfeld , G. McVicker , F. H. Gage , B. Ren , C. K. Glass , Science 2019, 366, 1134. 31727856 10.1126/science.aay0793 PMC7028213

[70] A. R. Quinlan , I. M. Hall , Bioinformatics 2010, 26, 841. 20110278 10.1093/bioinformatics/btq033 PMC2832824

[71] J. Vijay , M. F. Gauthier , R. L. Biswell , D. A. Louiselle , J. J. Johnston , W. A. Cheung , B. Belden , A. Pramatarova , L. Biertho , M. Gibson , M. M. Simon , H. Djambazian , A. Staffa , G. Bourque , A. Laitinen , J. Nystedt , M. C. Vohl , J. D. Fraser , T. Pastinen , A. Tchernof , E. Grundberg , Nat. Metab. 2020, 2, 97. 32066997 10.1038/s42255-019-0152-6 PMC7025882

[72] X. D. Liao , N. Sharma , F. Kapadia , G. J. Zhou , Y. Lu , H. Hong , K. Paruchuri , G. H. Mahabeleshwar , E. Dalmas , N. Venteclef , C. A. Flask , J. Kim , B. W. Doreian , K. Q. Lu , K. H. Kaestner , A. Hamik , K. Clement , M. K. Jain , J. Clin. Invest. 2011, 121, 2736. 21670502 10.1172/JCI45444 PMC3223832

[73] N. A. Jager , J. Westra , R. Golestani , G. M. van Dam , P. S. Low , R. A. Tio , R. H. Slart , H. H. Boersma , M. Bijl , C. J. Zeebregts , J. Nucl. Med. 2014, 55, 1945. 25359878 10.2967/jnumed.114.143180

