# Supplementary material for: DeCOOC Deconvoluted Hi‐C Map Characterizes the Chromatin Architecture of Cells in Physiologically Distinctive Tissues
Source: Adv Sci (Weinh). 2023 Jul 28;10(27):2301058. doi: 10.1002/advs.202301058 (PMC10520690; doi:10.1002/advs.202301058)
Supplement: Supplementary file 1 — Supporting Information [file ADVS-10-2301058-s003.pdf]

ADVANCED SCIENCE
Open Access

Supporting Information

for *Adv. Sci.*, DOI 10.1002/advs.202301058

DeCOOC Deconvoluted Hi-C Map Characterizes the Chromatin Architecture of Cells in Physiologically Distinctive Tissues

Junmei Wang, Lu Lu, Shiqi Zheng, Danyang Wang, Long Jin, Qing Zhang, Mingzhou Li* and Zhihua Zhang**

SUPPORTING INFORMATION

DeCOOC deconvoluted Hi-C map characterizes the chromatin architecture of cells in physiologically distinctive tissues

Junmei Wang[1,2,†], Lu Lu[3,4,†], Shiqi Zheng[1,2], Danyang Wang[1,2,5], Long Jin[3,4], Qing Zhang[1]*, Mingzhou Li[3,4]* and Zhihua Zhang[1,2]*

1 CAS Key Laboratory of Genome Sciences and Information, Beijing Institute of Genomics, Chinese Academy of Sciences and China National Center for Bioinformation, Beijing, 100101, China.

2 School of Life Science, University of Chinese Academy of Sciences, Beijing, 100049, China.

3 Livestock and Poultry Multiomics Key Laboratory of Ministry of Agriculture and Rural Affairs, College of Animal Science and Technology, Sichuan Agricultural University, Chengdu, 611130, China.

4 Animal Breeding and Genetics Key Laboratory of Sichuan Province, Institute of Animal Genetics and Breeding, Sichuan Agricultural University, Chengdu, 611130, China.

5 Sars-Fang Centre & MOE Key Laboratory of Marine Genetics and Breeding, College of Marine Life Sciences, Ocean University of China, Qingdao, 266100, China.

Contents:

Supplementary Text
Figure S1 to S16
Supplementary Tables 2 and 3, 5 to 7
Legends for Supplementary Tables 1 and 4
Reference

*To whom correspondence should be addressed. Tel: +86-10-84097249; Email: zhangzhihua@big.ac.cn(Z.Z.)

Correspondence may also be addressed to Mingzhou Li. Tel: +86-28-86290962; Email: mingzhou.li@sicau.edu.cn

[†]The authors wish it to be known that, in their opinion, the first two authors should be regarded as Joint First Authors.

Supplementary Text

Preprocessing for scHi-C datasets. For the mCC dataset[1], the authors processed a total of 2924 single F1 hybrid 129 × Castaneus mESCs grown in 2i media, and we chose 1992 cells that passed stringent quality control filters for further analysis in this study. We downloaded contact maps of the 1992 cells from https://github.com/tanaylab/schic2 and converted them into genomic coordinates using the R package misha (v. 4.0.6). We mapped genomic coordinates of all cells into restriction sites of the DpnII enzyme and obtained contacts whose format could be conveniently processed by Juicertools during the follow-up analysis. These cells were sorted into four phases of the mESC cell cycle: G1, Early_S, late_S_G2 and mitotic (pre-M and postM).

The HFC dataset[2] harbors 4238 single cells, including 14 human brain prefrontal cortex cell types, as follows: Astro (astrocyte), Endo (endothelial cell), MG (microglia), MP (nonneuronal cell type), ODC (oligodendrocyte), OPC (oligodendrocyte progenitor cell), excitatory neuron subtypes (L23, L4, L5 and L6), inhibitory neuron subtypes (Pvalb, Sst, Ndnf, and Vip). This dataset has a large number of sequencing contacts and a fairly high average interaction frequency per cell. The data were downloaded from Gene Expression Omnibus (GSE130711) and https://salkinstitute.app.box.com/s/fp63a4j36m5k255dhje3zcj5kfuzkyj1. To mimic the general sequencing depth of bulk samples at 500 kb resolution, we downsampled contacts of each single cell at a ratio of 0.1 to further build *in silico* bulk Hi-C, while integrated raw scHi-C contacts were taken as the basis for bulk Hi-C contacts of pure cell lines.

Calculation of the predicted results for test data. Five-fold cross-validation (80%-20% split for training samples) was used to train the model. Consequently, the model could be set with five sets of hyperparameters to predict the test dataset.

Using m_i to represent the input matrix for one bulk Hi-C sample (expressed as B) from the test dataset and $\hat{\boldsymbol{y}}_{j\_m_i} (1 \leq j \leq 5)$ to denote the predicted cell proportions from the jth set of parameters for c_i, the predicted cell proportions ($\hat{\boldsymbol{y}}_{m_i}$) were determined by the average of all predictions for c_i, i.e.,

$$\hat{\boldsymbol{y}}_{m_i} = \frac{1}{5}\sum_{j=1}^{5} \hat{\boldsymbol{y}}_{j\_m_i}$$

The final predicted results ($\hat{\boldsymbol{y}}_B$) for B were defined as the average of the predicted values from two matrices (m_1 and m_2, different coordinates from the same bulk sample), i.e.,

$$\hat{\boldsymbol{y}}_B = \frac{\hat{\boldsymbol{y}}_{m_1} + \hat{\boldsymbol{y}}_{m_2}}{2}$$

Prepare input for deCOOC model

The rationale behind merging two matrices from two chromosomes is to mitigate the potential selection of an inappropriate chromosome in the model by introducing a level of hedging.

Generally, a larger number of features tends to enhance the performance of machine learning tasks. Consequently, we explored three potential strategies:

1. Utilizing a larger matrix from one chromosome.
2. Concatenating two matrices from one chromosome.
3. Concatenating two matrices from two chromosomes.

Through careful evaluation, we determined that strategy number 3, involving the combination of two matrices from different chromosomes, yielded the most favorable outcomes. In contrast, the first and second strategies demonstrated inferior performance compared to the deCOOC method, and were susceptible to the selection of chromosomes with limited predictability. Notably, performance variations were observed among different chromosomes, possibly associated with the specific cell types under investigation. Consequently, it was challenging to establish a universally optimal chromosome for all cell types.

Nevertheless, an intriguing finding emerged from our analysis. Despite chromosomes exhibiting suboptimal performance in intra-chromosomal tests, their combination from two different chromosomes resulted in substantially improved performance (Figure S2 below). Consequently, the random pairing of two chromosomes provided deCOOC with consistent performance across diverse cell types not encountered during the training phase.

Brief introduction for other algorithms.

CIBERSORT (CS)[3] is a cell deconvolution algorithm developed on the basis of GEPs and linear support vector regression. CS is widely used to estimate cell type proportions in tissue samples with reference GEPs. Cell composition estimations in this study were performed using this algorithm (veltenlab/rnamagnet; https://github.com/veltenlab/rnamagnet/). For all deconvolutions with CS, we used the CIPs generated according to the section subtitled "Generation of reference matrices for the deconvolution", and we used bulk Hi-C data normalized with KR as bulk data subject to deconvolution, each column of which represented one sample. We used default settings for all CS parameters.

CDSeq[4], an unsupervised data mining tool, uses only RNA-seq data from bulk tissue samples to simultaneously estimate both cell mixing proportions and cell type-specific GEPs. Cell composition estimations in this article were completed with the R package CDSeq, v1.0.8. We used bulk data (same as CS) as input and CIPs as "reference_gep" param. We set param "cpu_number" as 5 and all other parameters as default settings for all deconvolutions.

DeconRNAseq[5] is a statistical framework for the deconvolution of heterogeneous tissue samples based on mRNA-seq data. It adopts a globally optimized nonnegative decomposition algorithm through quadratic programming to estimate the mixing proportions rigorously. It

requires input in the form of two R data frames that contain normalized Hi-C data from pure tissues and the bulk samples to be analyzed. Thus, we set a data frame with CIPs for param "refExpr" and another data frame with bulk hic of samples for "geneExpr". Estimations of cell proportions were completed with the R packages ADAPTS 1.0.6 and DeconRNAseq, v1.28.0.

Dtangle[6] is a simple and fast method to compute deconvolutions. It requires cell type-specific gene expression profiles as external knowledge. dtangle's approach robustly fits the model using log-transformed data and thus sets it apart from other deconvolution methods, and we made use of log-scale (base 2) bulk Hi-C and CIPs for estimating mixing proportions by dtangle accordingly. We completed the deconvolutions of dtangle using the R package dtangle, v2.0.9.

Fast and Robust DEconvolution of Expression Profiles (FARDEEP)[7] is a machine learning tool used to enumerate immune cell subsets from whole tumor tissue samples. FARDEEP utilizes an adaptive least trimmed square to automatically detect and remove outliers before estimating cell compositions, and it provides an estimate of relative percentages by normalizing absolute abundance to 1. We used the FARDEEP package, version 1.0.1, with "permn" set as 10 and default values for other parameters.

NNLS (implemented the Lawson-Hanson algorithm for nonnegative linear least squares)[8] solves the least squares problem $min\|Ax - b\|_2$ with the constraint $x \geq 0$, where $x \in R^n$, $b \in R^m$, and A is an $m \times n$ matrix. We used the 'nnls' function from the 'nnls' package v1.4 to accomplish cell type estimation of the samples and obtained the final proportions satisfying the sum-to-one constraint by scaling the results.

ssKL[9], a semisupervised NMF algorithm that minimizes the generalized Kullback–Leibler divergence, uses a set of known marker genes for each cell type to simultaneously estimate both the cell-specific signature and mixture proportion matrices. Similarly, the digital sorting algorithm (DSA)[10] also utilizes marker genes to estimate cell type proportions in a mixed tissue. We conducted deconvolution on bulk Hi-C samples mixed with only two cell types by these two methods, and they were implemented as part of the R packages CellMiX (v1.6.2) and DSA (v1.0) separately. Parameter "log" was set *False* for the two methods, and "sscale" was set *Fasle* for ssKL.

The two-step Hi-C UNsupervised DEconvolution appRoach (THUNDER)[11] is dedicated to the Hi-C deconvolution published recently. It consists of two steps: a feature selection step and deconvolution. Feature selection, as the first step, identifies informative bin pairs, which are genomic loci with informative contact frequencies across cell types. Deconvolution, as the second step, is performed based on the informative bin-pairs identified from the first step. Deconvolution was achieved by the 'run_thunder' function in the R package thundeR (v0.0.6), where the parameter "itter" was set as 20.

The predicted proportions from THUNDER do not correspond directly to real cell types. Therefore, to match the predicted proportion for each cell type, predictions with minimal RMSE compared to the proportions of a specific cell type would be defined as the predictions for that cell type, i.e.,

$$\min\left(\mathrm{RMSE}(\hat{\boldsymbol{p}}_c, \boldsymbol{p}_{c_1}), \mathrm{RMSE}(\hat{\boldsymbol{p}}_c, \boldsymbol{p}_{c_2})\right),$$

where $\hat{\boldsymbol{p}}_c$ denotes the predicted proportions of one cell type to be assigned a cell type label, and $\boldsymbol{p}_{c_1}$ and $\boldsymbol{p}_{c_2}$ indicate the true proportions contained in different samples for cell types one and two, respectively.

Fine-tuning for the HFC dataset

The deCOOC model achieves its generalization capability by employing an additional fine-tuning process to handle samples with previously unseen cell types. In this particular case, we randomly selected 11 out of 14 cell types from the HFC dataset and generated simulated bulk Hi-C mixture samples to train the model. Subsequently, we created a total of 160 bulk Hi-C mixture samples, wherein 2 to 3 cell types were not encountered during the training stage. These 160 samples were divided into two groups: one consisting of 20 samples for fine-tuning and the other containing 140 samples for testing.

To fine-tune the model, we specifically updated the last two layers of the model using the 20 randomly selected samples for fine-tuning. Following this fine-tuning process, we employed the fine-tuned model to directly predict the remaining 140 test samples. We evaluated the model's prediction performance using the Concordance Correlation Coefficient (CCC) and Root Mean Square Error (RMSE). The median CCC was found to be 0.968 (with an interquartile range [IQR] of 0.952-0.984), while the RMSE was 0.037 (with an IQR of 0.018-0.051) (see Figure S14A).

Additionally, we obtained a pre-trained model by combining the training data from the mCC dataset with our current HFC training dataset. By fine-tuning this model using the same samples, we achieved significantly improved results compared to the pre-trained model trained solely using the HFC training dataset (see Figure S14B).

Heatmap analysis with SHAP values. Values (greater than the 90th percentile) in the SHAP map of each predicted cell type were reserved, as well as the genomic loci of these values. These were then analyzed for important cell type-specific interaction characteristics in predictions.

Cell-type SHAP values construction for joint analysis with ATAC data. For each cell type, the SHAP values of all interactions were defined as the average of SHAP values across all matrices from bulk data (in which this cell type accounted for more than 90%).

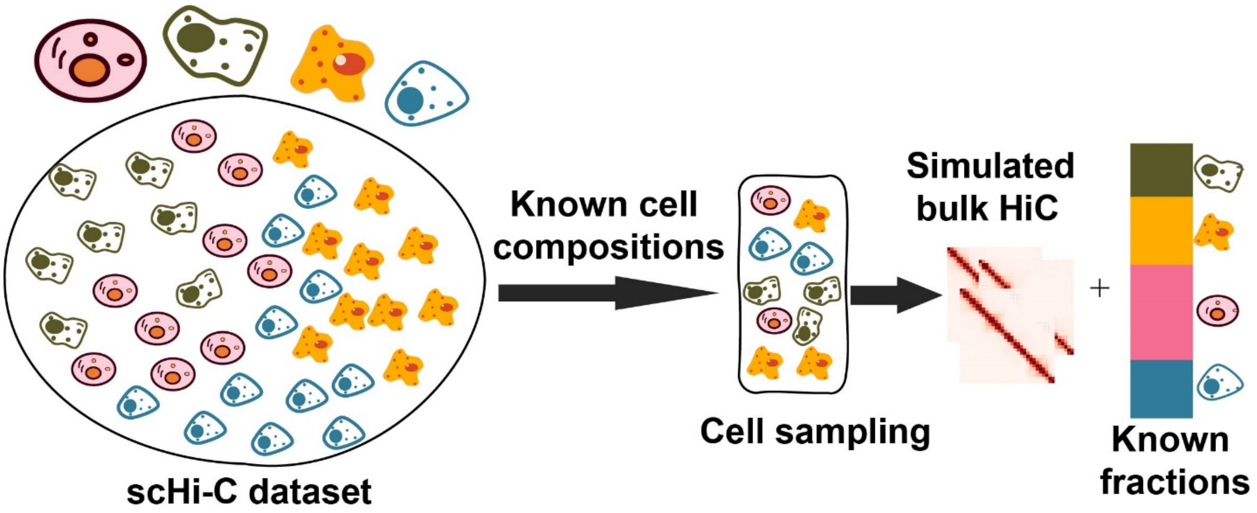

Figure S1. Overview of simulated bulk Hi-C data generation. *In silico* bulk Hi-C samples were generated by randomly sampling single cells from the scHiC dataset according to predefined cell type compositions. Each shape represents one cell type.

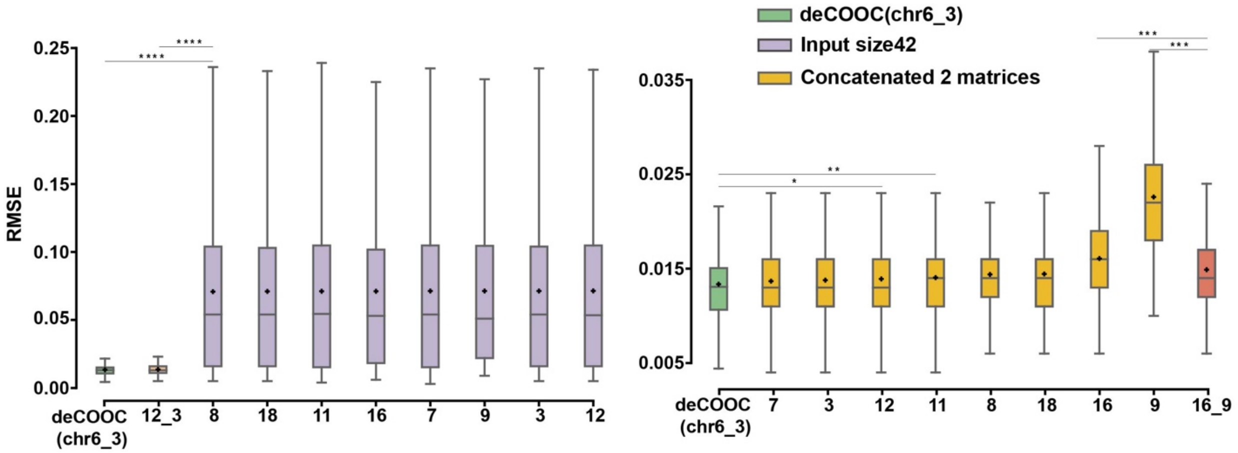

Figure S2. Comparison of RMSE values from deCOOC and the models trained using Hi-C matrices from larger matrix or concatenated matrices from same chromosome.

In this analysis, we focused on the HFC data and evaluated the performance of deCOOC comparing to the models trained with two different strategies: (1) a larger matrix (42x42, the left panel) and (2) concatenated matrices from same chromosome (30x60, the right panel). Eight chromosomes were randomly selected for comparison. The order of the chromosomes in the figure corresponds to the performance of the models trained with the larger matrix or concatenated matrices. Additionally, we included an extra model trained with concatenated matrices from the two worst-performing chromosomes (chr 12, 3 and chr 16, 9, for left and right panel, respectively) for further comparison. The p-values were calculated using a one-sided t-test. *P< 0.05, **P< 0.01, ***P< 0.001, and ***P<0.0001.

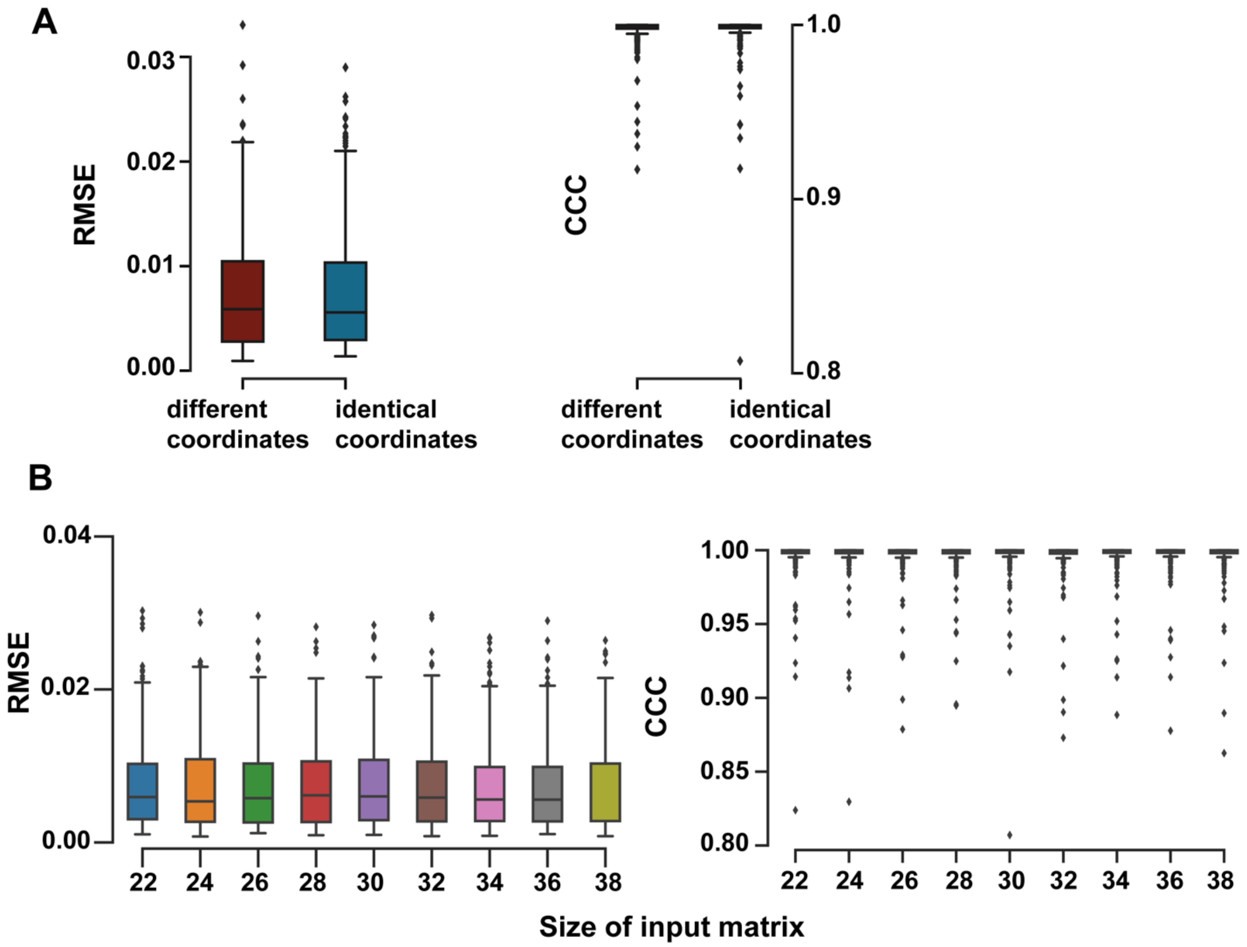

Figure S3. Sensitivity of the deconvolution performance of deCOOC to the model's input. **A**) RMSE and CCC values when two matrices in the input were in different or identical coordinates. No significant difference was observed between these two settings (p>0.2). **B**) Comparison of RMSE and CCC values from deCOOC with different sizes of input matrix, e.g., '22' indicates that the size of square-like matrix from one chromosome is 22 bins. No significant difference was noted between the sizes shown for both RMSE and CCC values. *P* values were calculated using a two-sided Wilcoxon signed-rank test.

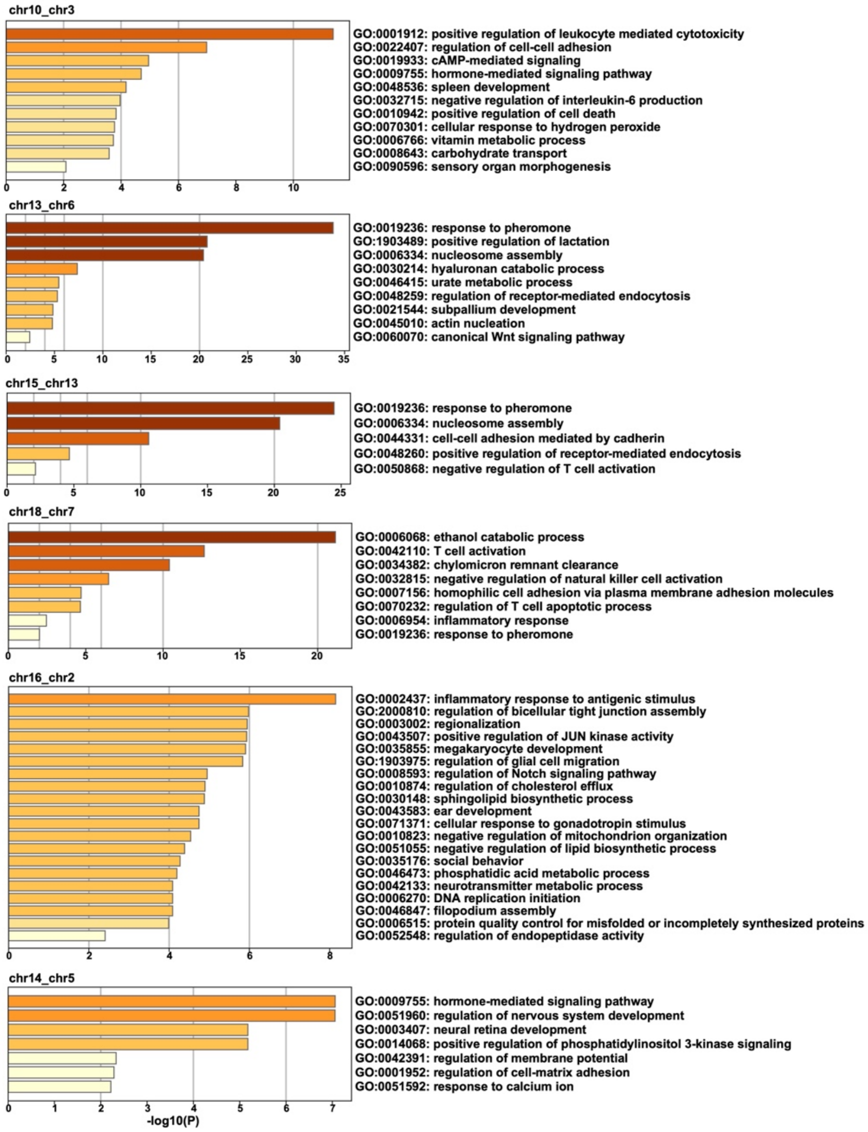

Figure S4. The figure presents the results of the GO enrichment analysis conducted on the model trained using various chromosome combinations. The analysis aimed to identify specific regions (13-28MB on each two chromosomes) that exhibited high positive SHAP values (>90%). The gene ontology terms were observed for the G1 cell type in the mCC example shown in Figure 4C. The figure demonstrates that the combination of chr10 and chr3 achieved the highest accuracy, with a median Root Mean Square Error (RMSE) of 0.0057 (with an interquartile range [IQR] of 0.003-0.0106) and a median Concordance Correlation Coefficient (CCC) of 0.9994 (with an IQR of 0.9980-0.9999). Conversely, the lowest accuracy was observed in the chr16 and chr2 combination, with a median RMSE of 0.0067 (with an IQR of 0.0031-0.0114) and a median CCC of 0.9993 (with an IQR of 0.9978-0.9999). We acknowledge that subtle differences can arise when selecting interaction matrices from chromosomes rich in relevant genes. However,

such selections can enhance the model's prediction accuracy based on prior knowledge of cell types.

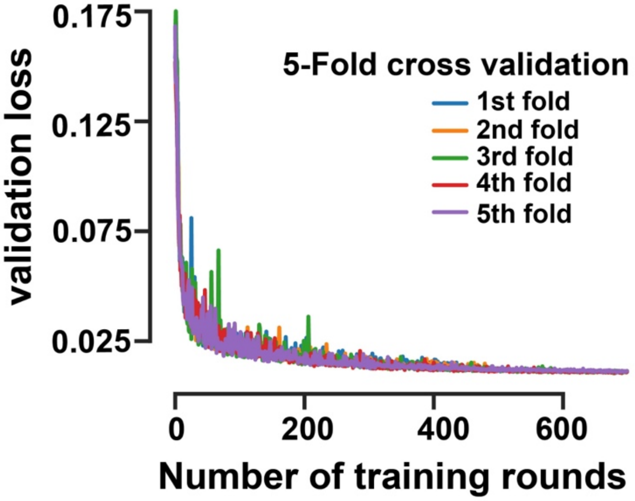

Figure S5. Validation loss measured by RMSE on validation data during the training process for five-fold cross-validation.

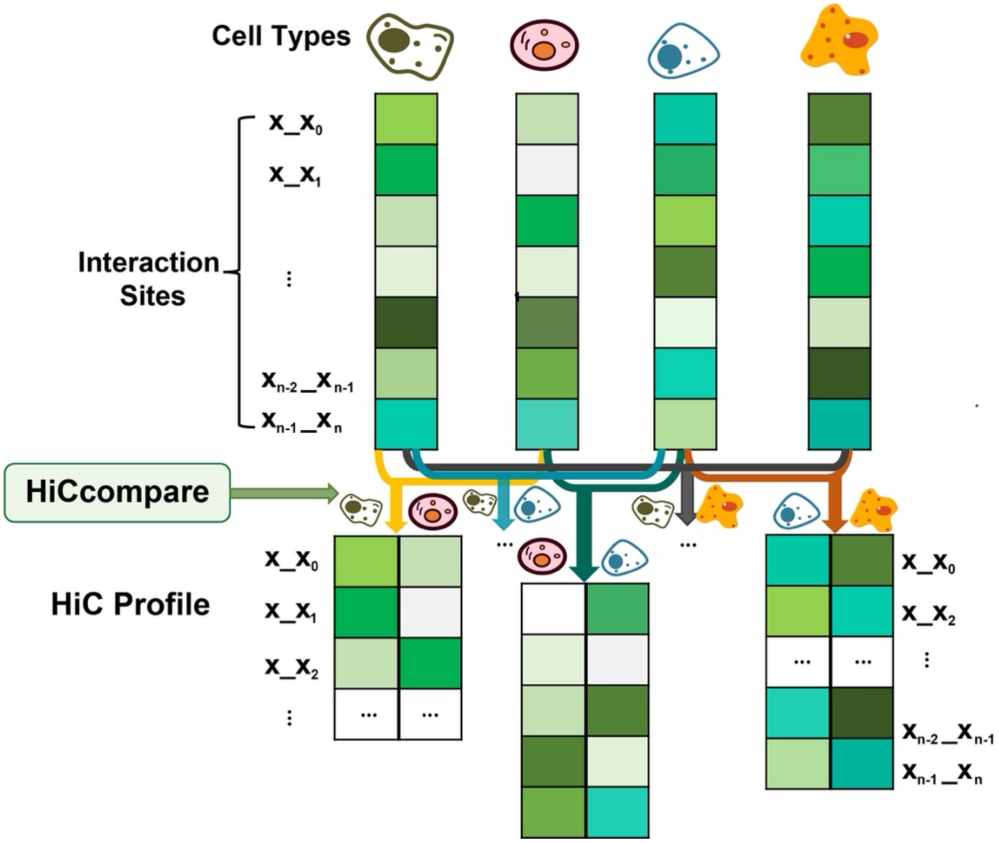

Figure S6. **Workflow for the generation of cell type-specific reference matrices.** For any two cell types, HiCcompare was applied to the Hi-C matrices of the same chromosome for any two cell types. Differential interactions of each chromosome for any two cell types were site-to-site stitched together as these two cell type-specific Hi-C profiles.

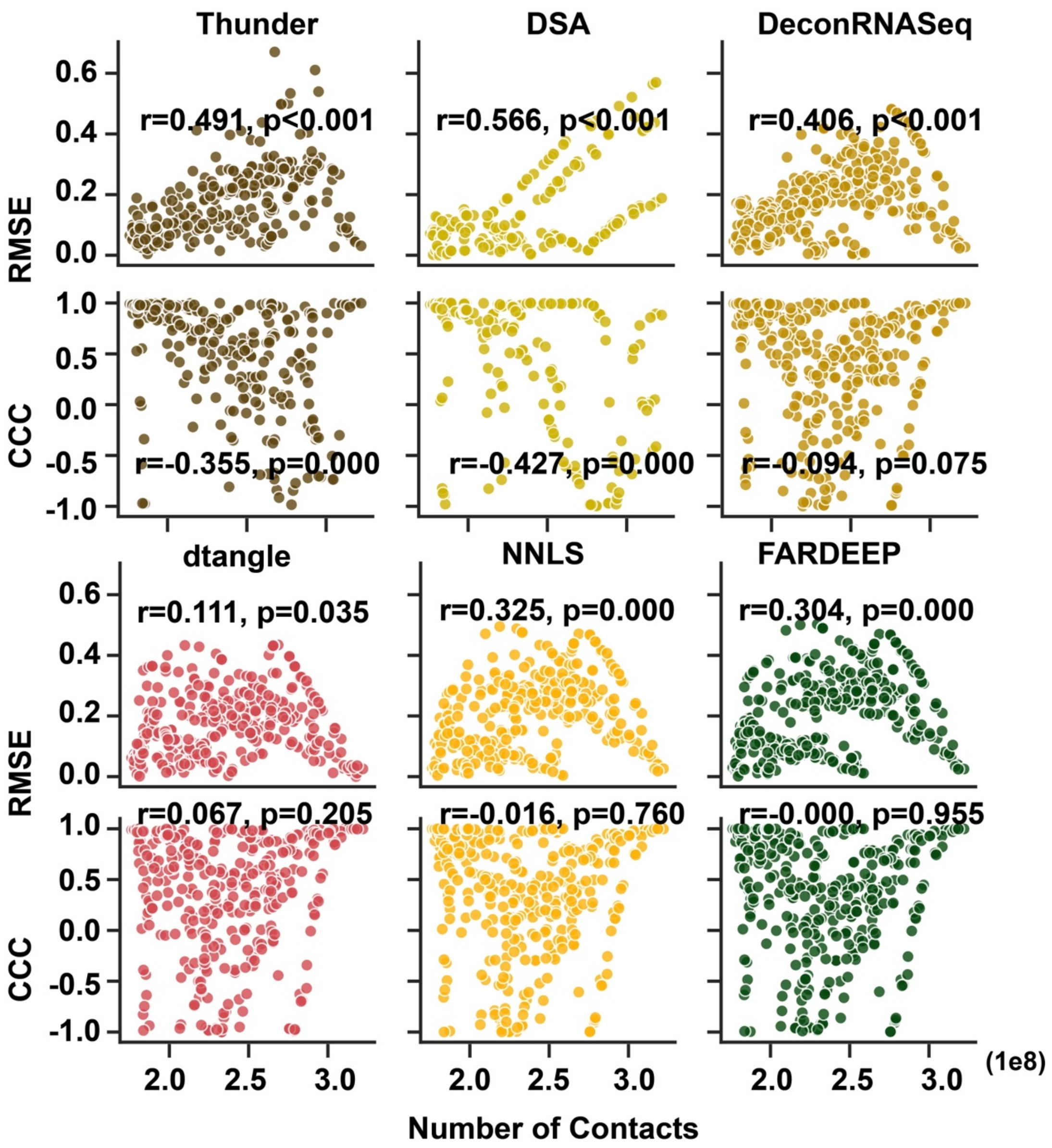

Figure S7. Correlation between prediction performance and the number of contacts for different methods. Scatterplots of RMSE (and CCC) values and the number of Hi-C interaction contacts of the simulated mCC test dataset for Thunder, DSA, DeconRNASeq, dtangle, NNLS and FARDEEP. Pearson correlation coefficients and p values are given above the plots.

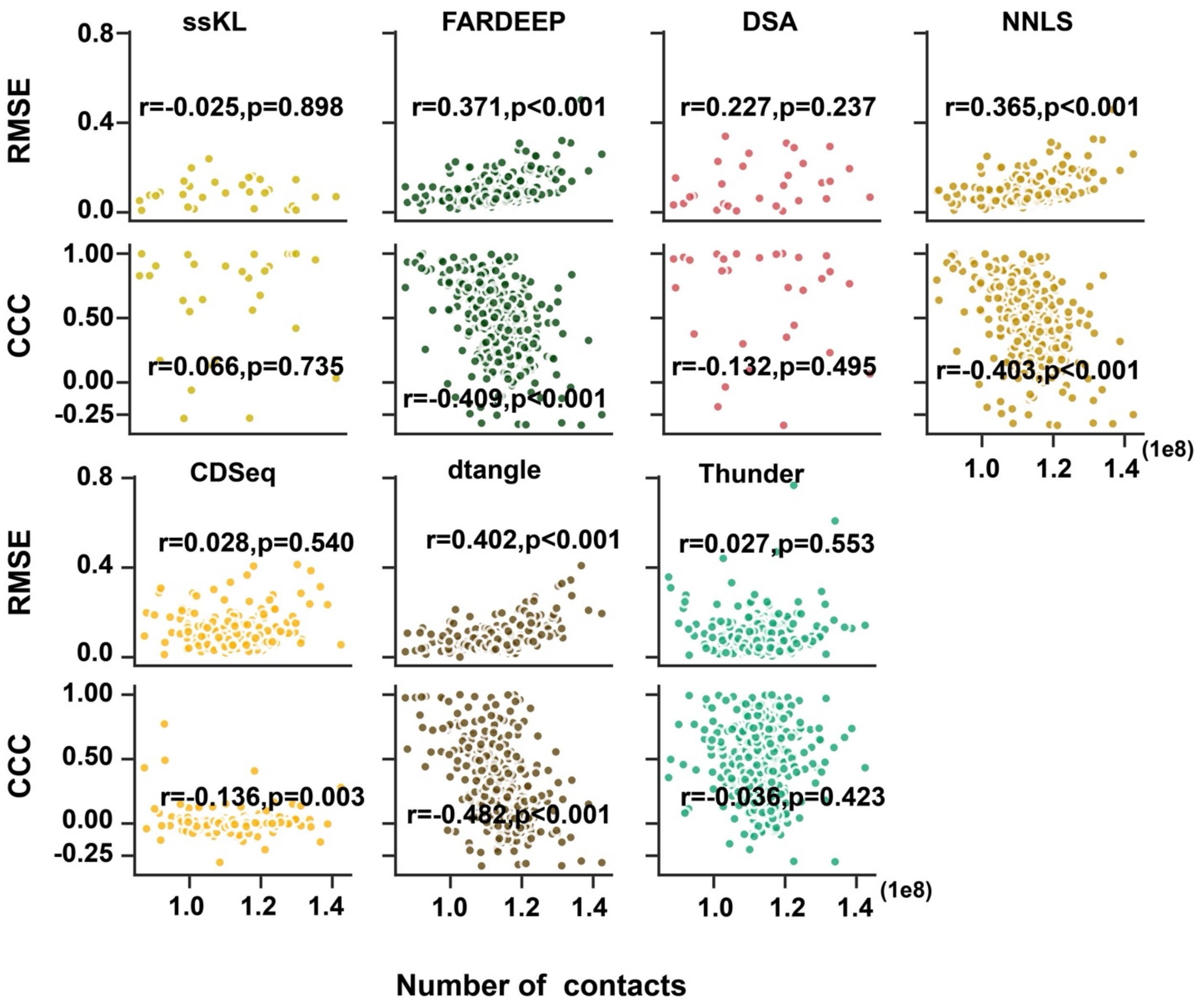

Figure S8. Scatterplots of RMSE (and CCC) values and the number of Hi-C interaction contacts of the simulated HFC test dataset for ssKL, FARDEEP, DSA, NNLS, CDSeq, dtangle, and Thunder. Pearson correlation coefficients and p values are given above the plots.

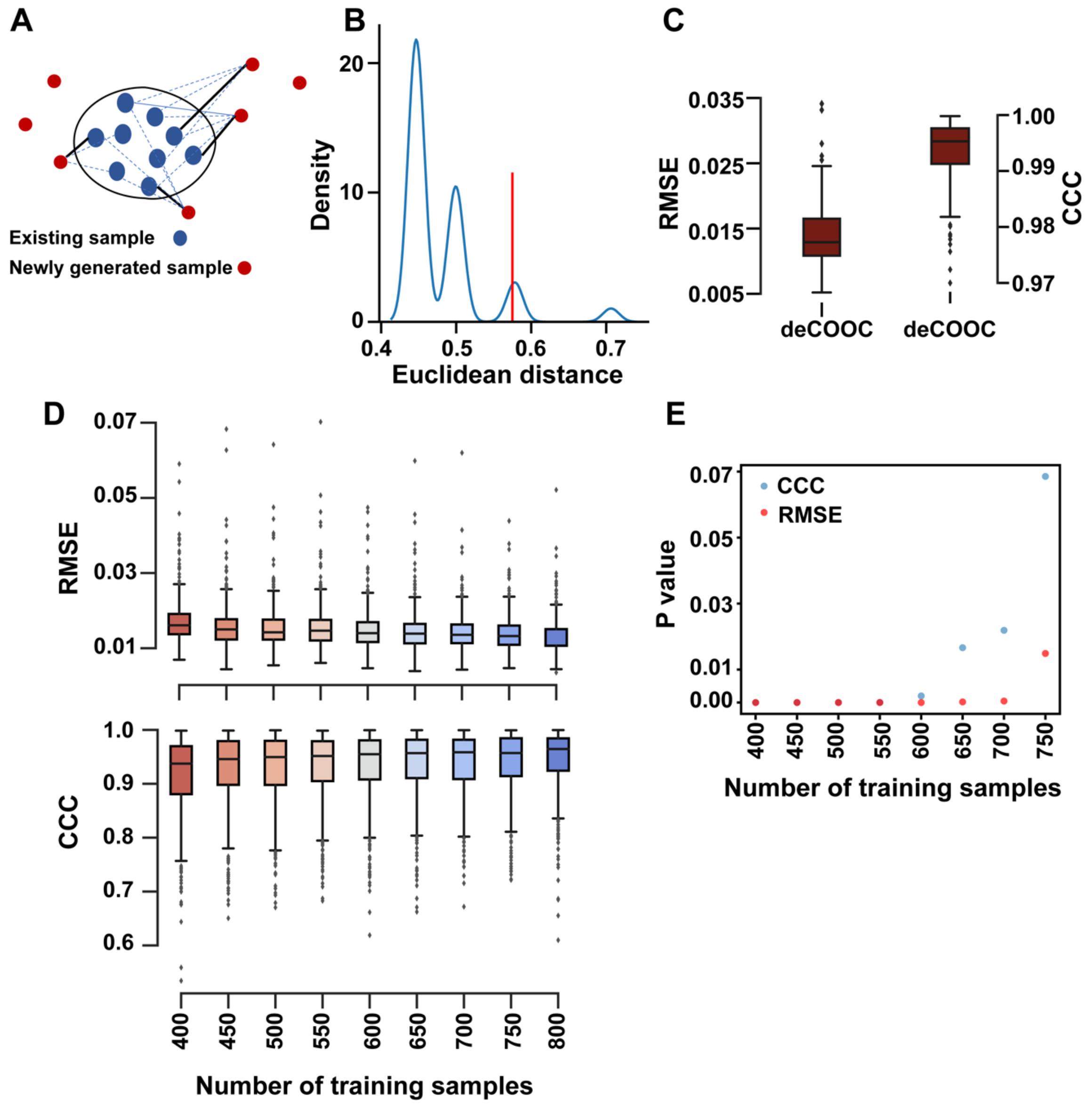

Figure S9. Validation of the generalization ability of deCOOC. A) Generating new bulk samples and calculating the Euclidean distance between newly generated samples and existing samples. The solid line indicates the shortest distance of each new sample to the existing samples. **B**) Distance distribution of newly generated samples. Samples that had distance values greater than the value (e.g., 0.56) indicated by the red line were defined as unseen samples far from existing samples. **C**) Prediction performance of deCOOC on 200 randomly selected unseen samples far from existing samples. **D**) Overview of deconvolution performance on the same test dataset, while the model was trained using different numbers of training samples. **E**) One-sided Wilcoxon signed-rank test p values for performance (RMSE and CCC) comparison between the model trained with each number of training samples (less than 800) and 800 samples.

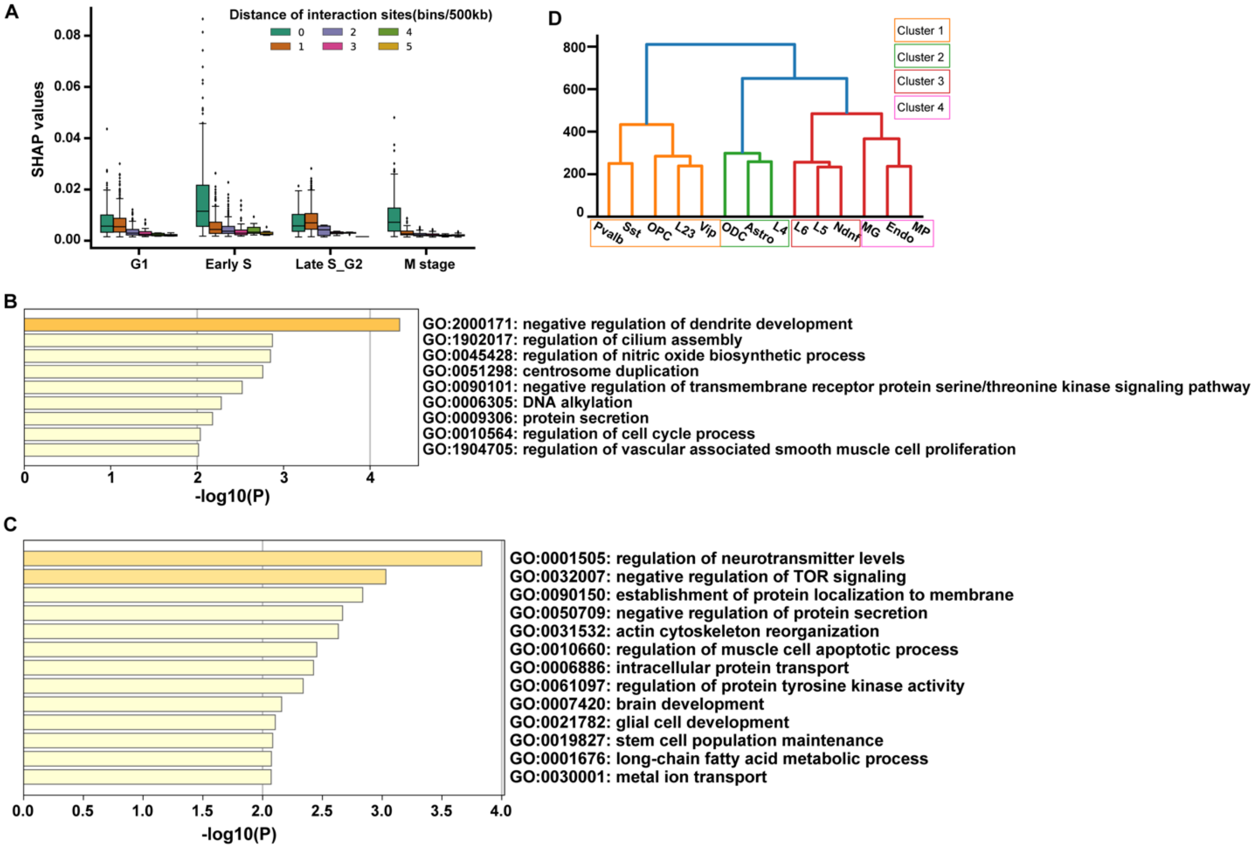

Figure S10. Analysis of SHAP values for model interpretation. A) Distribution of SHAP values among different genomic distances between interaction sites for four cell types in the mCC dataset. These SHAP values were based on cell-type SHAP values (Supplementary text). **B**-**C**) GO enrichment analysis for regions with high positive SHAP values (>=90%) from the heatmap in Fig. 4D. GO terms for the G1 cell type of the mCC example (in panel **B**) and astrocytes of the HFC example (in panel **C**). **D**) Hierarchical clustering based on SHAP values of each cell type for the HFC example shown in Fig. 4D. We obtained four clusters that were different from the categories shown in Fig. 4B.

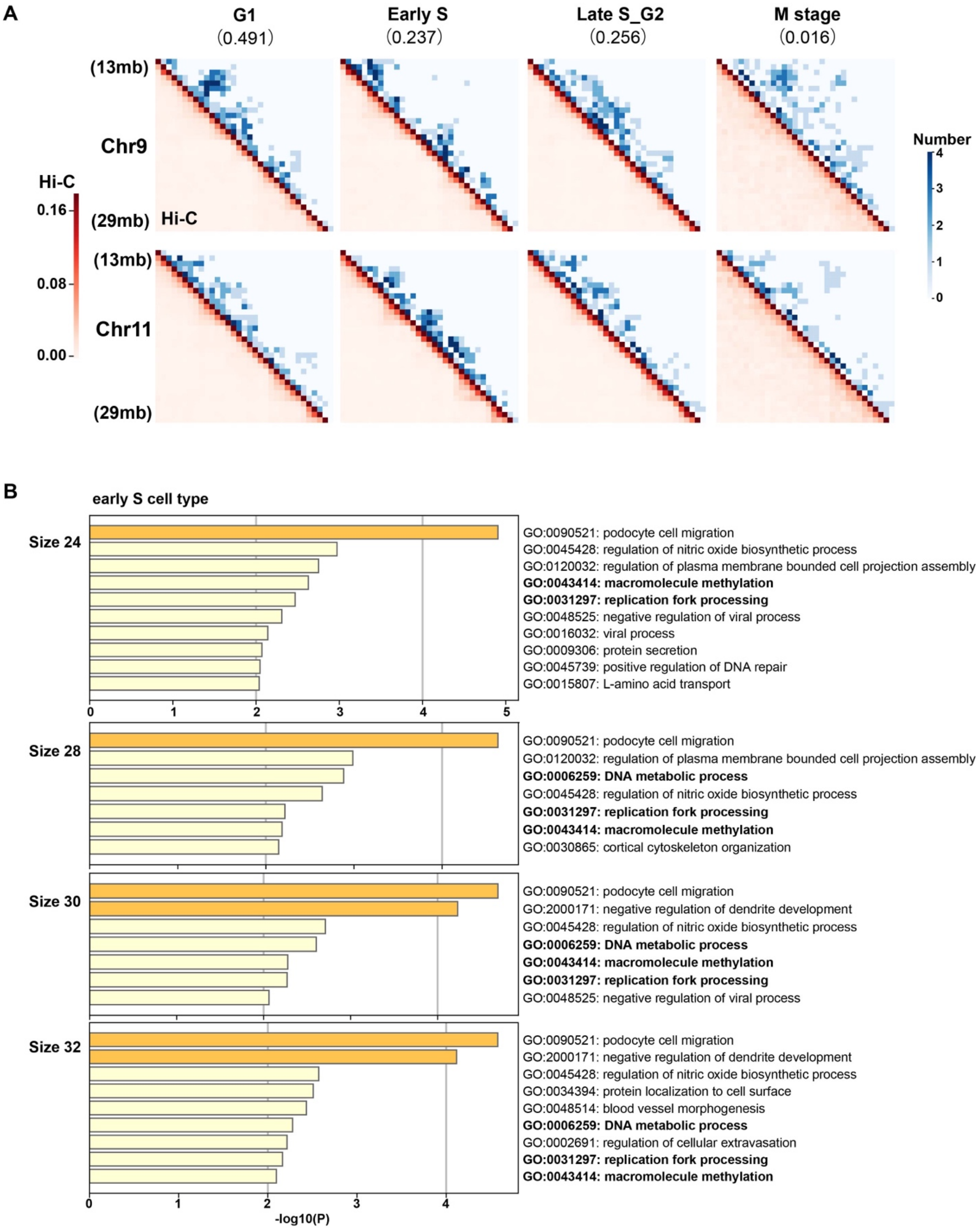

Figure S11. The interpretability of models trained with different input sizes (24, 28, 30, and 32).

A) Examples of paired Hi-C matrices (lower left) and the distribution of high SHAP values (>90%) across bins (upper right) in four models trained with varying input sizes for each cell type of the mCC example. The pixels in the upright part of the matrices represent the number of times each bin has been considered as a contributor (i.e., belonging to the high (top 10%) SHAP values) across the four models of varying input sizes. B) GO enrichment analysis for genes located in the contributor regions, as demonstrated in panel A.

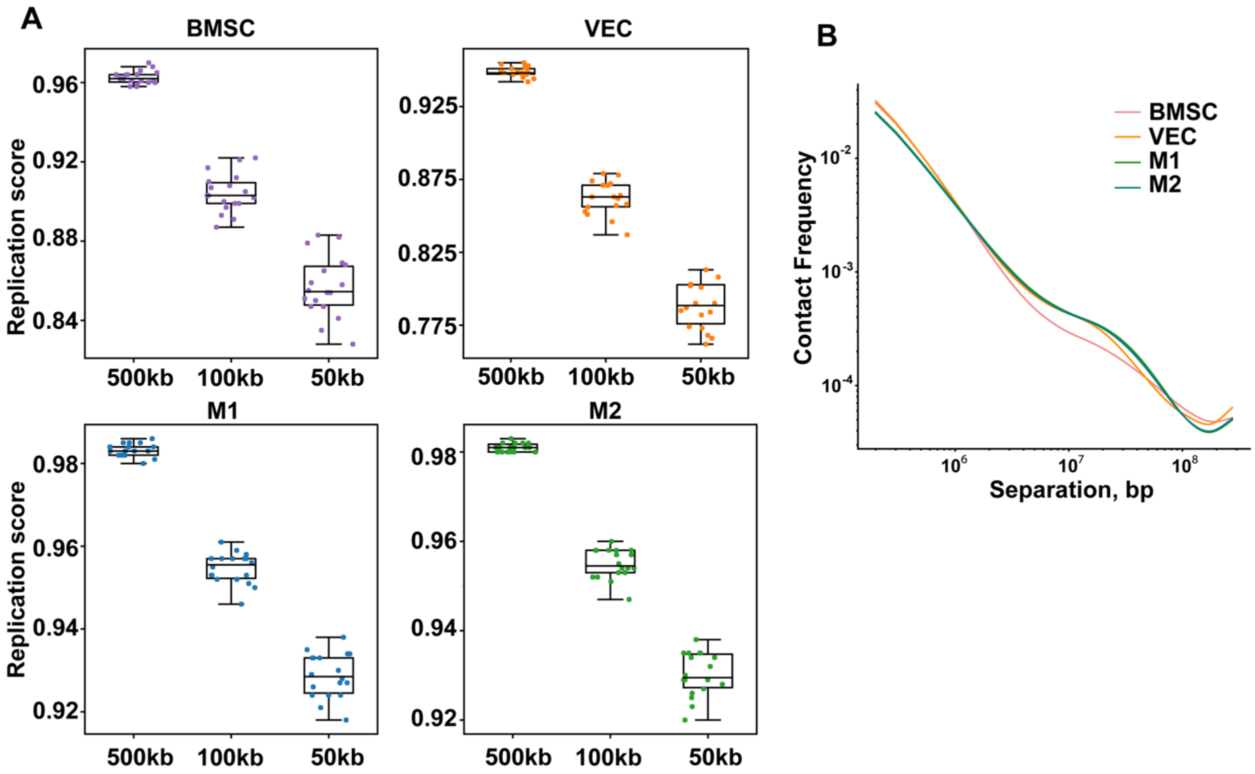

Figure S12. Quality of experimental *in situ* Hi-C data for four pig cell types. A) Replicate score of four pig cell types (500 kb resolution was used in this study). **B**) Distribution between contact frequency and genomic separation for four pig cell types.

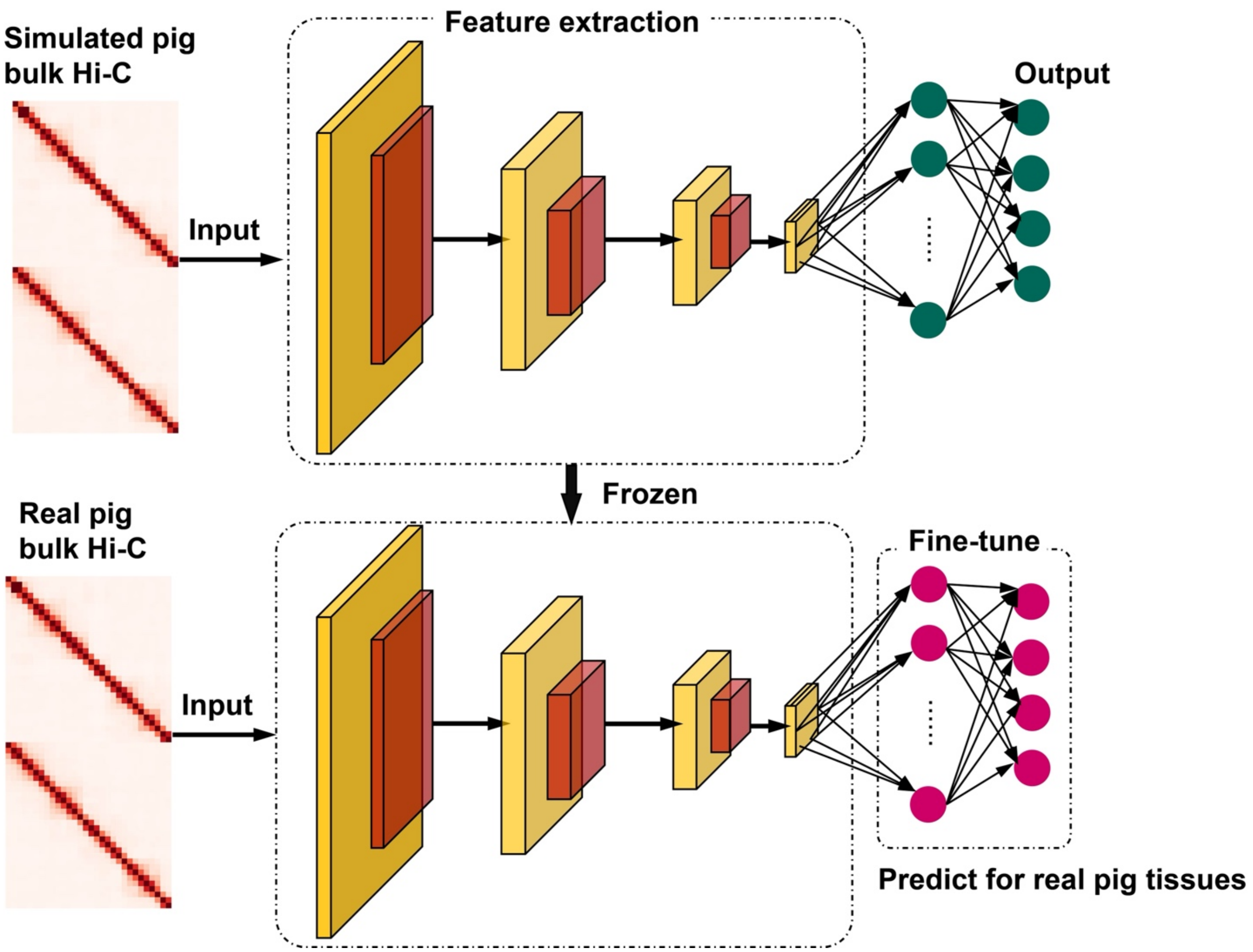

Figure S13. Overview of fine-tuning for real pig tissues. First, deCOOC is pretrained using simulated pig bulk Hi-C data. Then, all layers, except fully connected dense layers, in the trained model are frozen so that their weights cannot be updated when fine-tuning. deCOOC will perform deconvolution on real pig tissues when it is fine-tuned using a small number of real pig tissues.

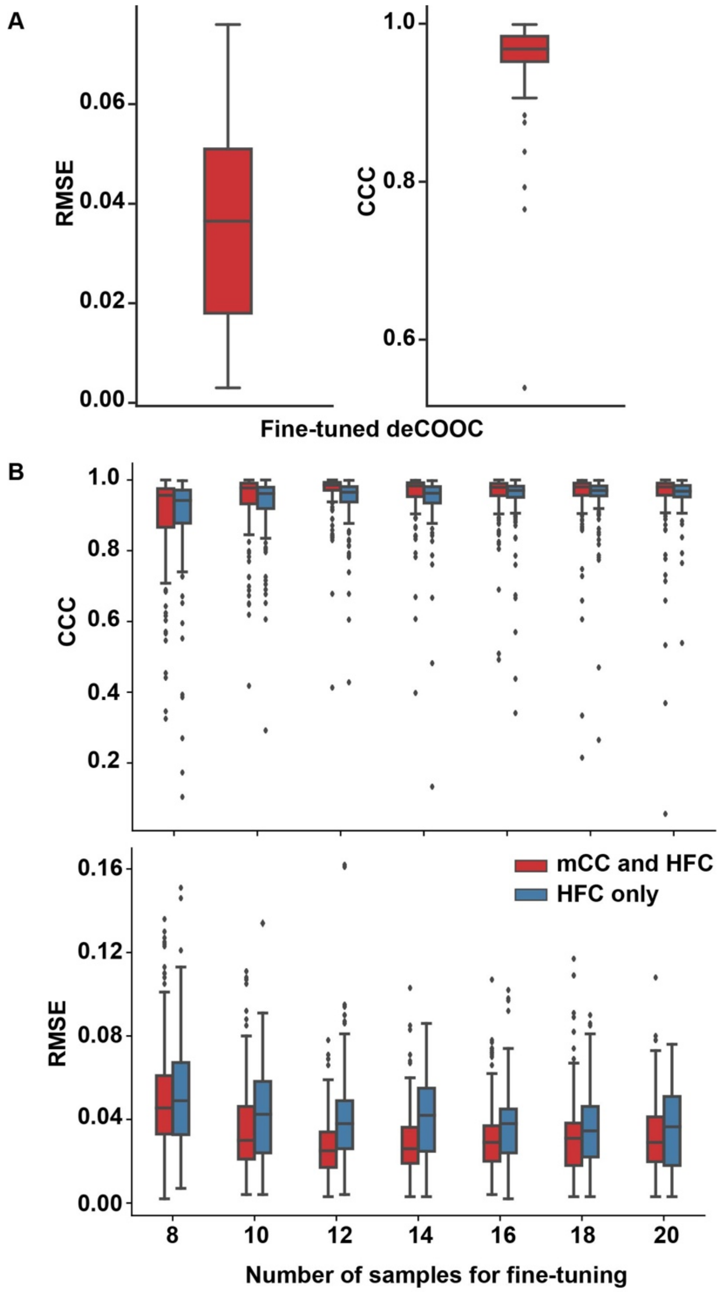

A
RMSE
0.00
0.02
0.04
0.06
CCC
0.6
0.8
1.0
Fine-tuned deCOOC
B
CCC
0.2
0.4
0.6
0.8
1.0
RMSE
0.00
0.04
0.08
0.12
0.16
mCC and HFC
HFC only
8
10
12
14
16
18
20
Number of samples for fine-tuning

Figure S14: An overview of the fine-tuning process for the HFC dataset. A) The Root Mean Square Error (RMSE) and Concordance Correlation Coefficient (CCC) performance of the fine-tuned deCOOC model on the HFC dataset. More detailed information can be found in the Supplementary text. B) An overview of the deconvolution performance for the fine-tuned deCOOC model. The model was fine-tuned on a pre-trained model that was initially trained using both the mCC and HFC datasets (indicated in red) or trained using the HFC dataset alone (indicated in blue). The x-axis represents the number of samples used for fine-tuning.

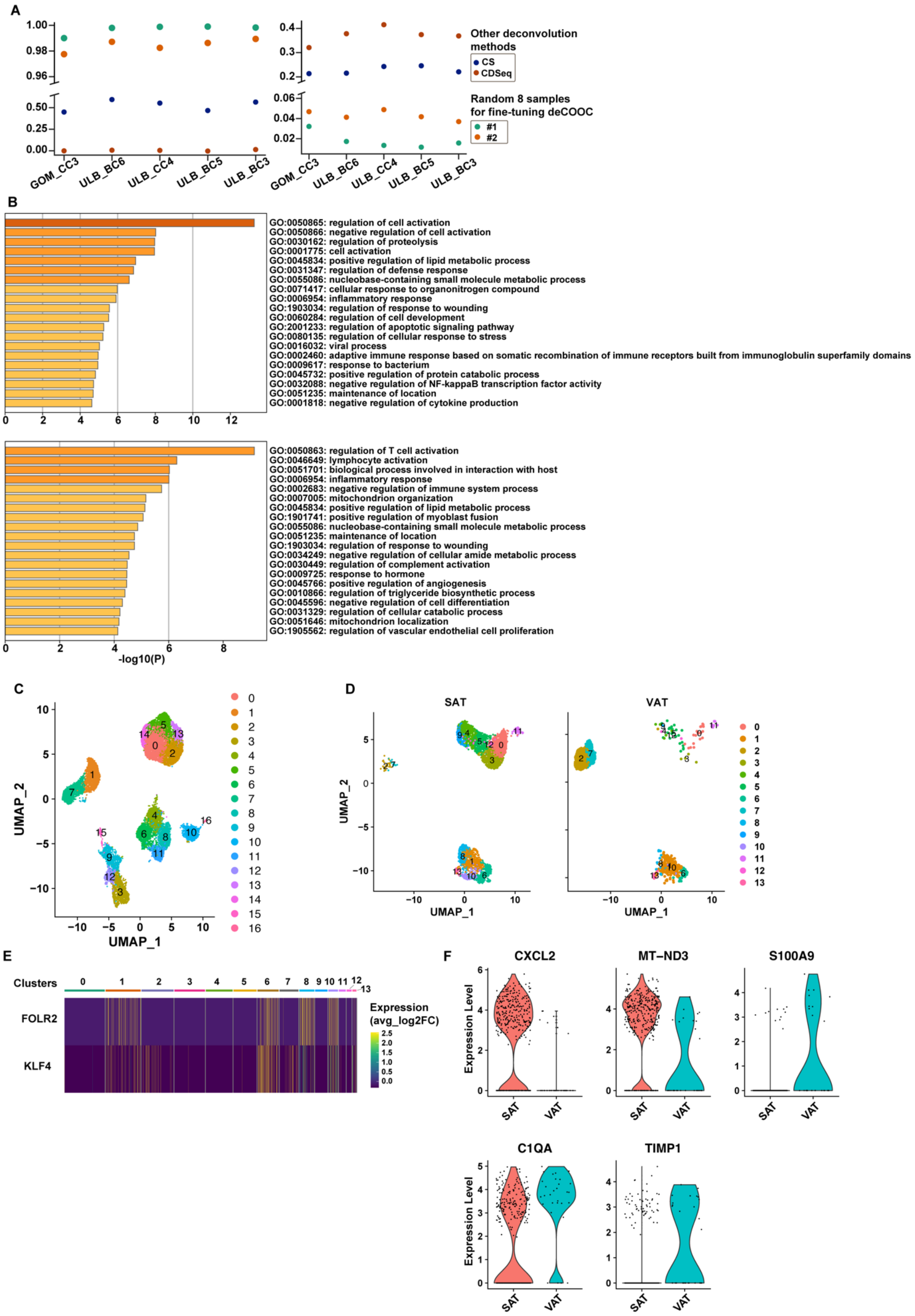
A
Other deconvolution methods
CS
CDSeq
Random 8 samples for fine-tuning deCOOC
#1
#2
GOM_CC3
ULB_BC6
ULB_CC4
ULB_BC5
ULB_BC3
B
GO:0050865: regulation of cell activation
GO:0050866: negative regulation of cell activation
GO:0030162: regulation of proteolysis
GO:0001775: cell activation
GO:0045834: positive regulation of lipid metabolic process
GO:0031347: regulation of defense response
GO:0055086: nucleobase-containing small molecule metabolic process
GO:0071417: cellular response to organonitrogen compound
GO:0006954: inflammatory response
GO:1903034: regulation of response to wounding
GO:0060284: regulation of cell development
GO:2001233: regulation of apoptotic signaling pathway
GO:0080135: regulation of cellular response to stress
GO:0016032: viral process
GO:0002460: adaptive immune response based on somatic recombination of immune receptors built from immunoglobulin superfamily domains
GO:0009617: response to bacterium
GO:0045732: positive regulation of protein catabolic process
GO:0032088: negative regulation of NF-kappaB transcription factor activity
GO:0051235: maintenance of location
GO:0001818: negative regulation of cytokine production
GO:0050863: regulation of T cell activation
GO:0046649: lymphocyte activation
GO:0051701: biological process involved in interaction with host
GO:0006954: inflammatory response
GO:0002683: negative regulation of immune system process
GO:0007005: mitochondrion organization
GO:0045834: positive regulation of lipid metabolic process
GO:1901741: positive regulation of myoblast fusion
GO:0055086: nucleobase-containing small molecule metabolic process
GO:0051235: maintenance of location
GO:1903034: regulation of response to wounding
GO:0034249: negative regulation of cellular amide metabolic process
GO:0030449: regulation of complement activation
GO:0009725: response to hormone
GO:0045766: positive regulation of angiogenesis
GO:0010866: regulation of triglyceride biosynthetic process
GO:0045596: negative regulation of cell differentiation
GO:0031329: regulation of cellular catabolic process
GO:0051646: mitochondrion localization
GO:1905562: regulation of vascular endothelial cell proliferation
-log10(P)
C
UMAP_1
UMAP_2
D
SAT
VAT
E
Clusters
FOLR2
KLF4
Expression (avg_log2FC)
F
CXCL2
MT−ND3
S100A9
C1QA
TIMP1
Expression Level

Figure S15. Analysis of model interpretation based on SAT and VAT. A) RMSE and CCC values of deconvolution on five real test tissues (x-axis) from the three deconvolution methods. deCOOC was fine-tuned using two groups of real tissues, both of which contain eight different real tissues. **B**) GO enrichment analysis for regions (10-85 mb in chr2 and chr3) with positive (>=90%) SHAP values (by "GradientExplainer" applied on fine-tuned deCOOC) when predicting M2 cell type in GOM (top panel) and ULB (bottom) tissue, respectively. **C**) Clustering results of 14,370 cells from the SVF derived from 17 nondiabetic adipose samples that underwent scRNA-seq, identifying 17 clusters**.** Cell populations (1, 4, 7, 8, 12 and 15) were classified as immune cells. **D**) Clustering results of 5000 immune cells from Figure S12B, identifying 14 subclusters (for subcutaneous adipose tissue (SAT) and visceral adipose tissue (VAT))**. E**) Expression of gene markers (FOLR2 and KLF4) for different clusters in Figure S12C. **F**) Differential expression on a log 2 scale of five genes for subcutaneous adipose tissue (SAT) and visceral adipose tissue (VAT).

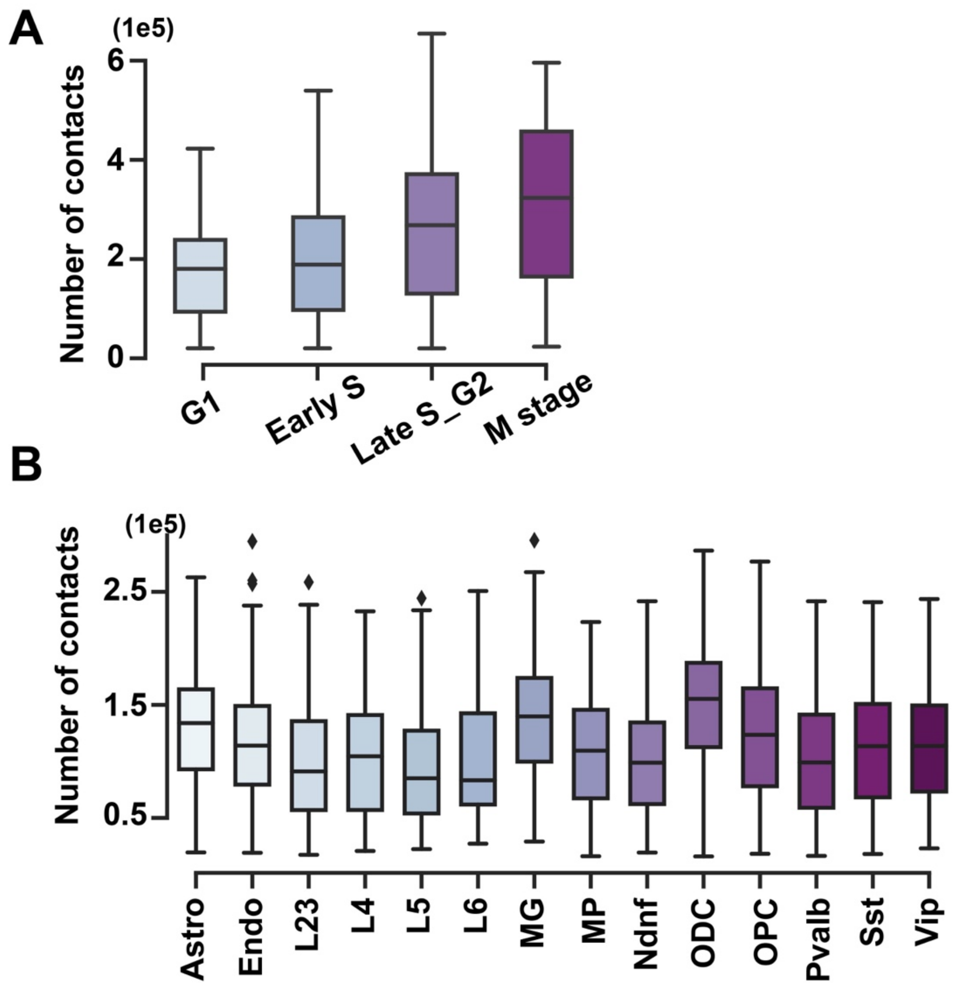

Figure S16. Distribution of the number of contacts for all existing single cells we used. A) Distribution of the number of contacts of each cell type in the mCC dataset. **B**) Distribution of the number of contacts for downsampled single cells of each type in the HFC dataset.

Supplementary Table 1. Performance of deCOOC trained with random combinations of two chromosomes.

(Refer Supplementary Excel file)

Supplementary Table 2. Overview of the number of combinations making up simulated bulk samples for the HFC dataset.

Number of cell types making up bulk samples	Number of Combinations
2	51
3	95
4	78
5	79
6	90
7	102
8	79
9	91
10	71
11	91
12	50
13	14
14	1

Supplementary Table 3. Brief summary of Hi-C data for four pig cell types.

	Adi	VEC	M1	M2
Sequenced Read Pairs	1,208,569,494	1,598,320,331	1,626,962,287	1,815,447,092
Alignable (Normal+Chimeric Paired)	1,037,309,964 (85.83%)	1,532,332,391 (95.87%)	1,464,309,294 (90.00%)	1,610,562,481 (88.71%)
Unique Reads	481,314,038 (39.83%)	518,063,661 (32.41%)	758,151,995 (46.60%)	777,571,403 (42.83%)
Hi-C Contacts (MAPX>=30)	335,509,963 (27.76%/69.71%)	420,171,962 (26.29%/81.10%)	603,964,544 (37.12%/79.66%)	601,217,666 (33.12%/77.32%)
Interchromosomal	48,208,499 (3.99%/10.02%)	19,627,316 (1.23%/3.79%)	75,632,545 (4.65%/9.98%)	86,913,922 (4.79%/11.18%)
Intrachromosomal	287,301,464 (23.77%/59.69%)	400,544,646 (25.06%/77.32%)	528,331,999 (32.47%/69.69%)	514,303,744 (28.33%/66.14%)
Short Range (<20Kb)	220,925,765 (18.28%/45.90%)	364,951,795 (22.83%/70.45%)	410,420,713 (25.23%/54.13%)	386,412,427 (21.28%/49.69%)
Long Range (>20Kb)	66,337,890 (5.49%/13.78%)	35,589,382 (2.23%/6.87%)	117,827,902 (7.24%/15.54%)	127,805,717 (7.04%/16.44%)

Supplementary Table 4. Data formation for real pig adipose tissues.
(Refer Supplementary Excel File)

Supplementary Table 5. Brief introduction of the mCC dataset, including the number of existing single cells and simulated single cells required for simulated Hi-C bulk for each cell type.

Type	Number of existing cells	Median contacts	Number of simulated single cells required
G1	404	180336	584
Early S	882	188947	106
Late S_G2	649	268336	339
M stage	57	323332	341

Supplementary Table 6. Brief introduction of the HFC dataset, including the number of existing single cells and simulated single cells required for simulated Hi-C bulk for each cell type.

Cell Type	Number of existing cells	Median contacts	Number of simulated single cells required
Astro	449	133890	304
Endo	205	113963	695
L23	551	91115	95
L4	131	104490	749
L5	180	85028	756
L6	86	83288	846
MG	422	139765	438
MP	100	109508	722
Ndnf	144	98840	569
ODC	1245	155319	0
OPC	203	123468	671
Pvalb	134	99061	630
Sst	217	113482	521
Vip	171	113596	793

Supplementary Table 7. Pairing downloaded ATAC-seq data with cell types for the HFC dataset.

Downloaded files	Matching cell type in HFC dataset
LHX2_optimal_peak_IDR_ENCODE.ATAC	Astro
NeuN_optimal_peak_IDR_ENCODE.ATAC	L23
Olig2_optimal_peak_IDR_ENCODE.ATAC	ODC
PU1_optimal_peak_IDR_ENCODE.ATAC	MG

References

[1] J. P. Zape, C. O. Lizama, K. M. Cautivo, A. C. Zovein *Cell Cycle* **2017**, *16*,1835-1847.
[2] D. S. Lee, C. Luo, J. Zhou, S. Chandran, A. Rivkin, A. Bartlett, J. R. Nery, C. Fitzpatrick, C. O'Connor, J. R. Dixon, J. R. Ecker *Nat Methods* **2019**, *16*,999-1006.
[3] A. M. Newman, C. L. Liu, M. R. Green, A. J. Gentles, W. Feng, Y. Xu, C. D. Hoang, M. Diehn, A. A. Alizadeh *Nat Methods* **2015**, *12*,453-7.
[4] K. Kang, Q. Meng, I. Shats, D. M. Umbach, M. Li, Y. Li, X. Li, L. Li *PLoS Comput Biol* **2019**, *15*,e1007510.
[5] T. Gong, J. D. Szustakowski *Bioinformatics* **2013**, *29*,1083-5.
[6] G. J. Hunt, S. Freytag, M. Bahlo, J. A. Gagnon-Bartsch *Bioinformatics* **2019**, *35*,2093-2099.
[7] Y. N. Hao, M. Yan, B. R. Heath, Y. L. Lei, Y. Y. Xie *Plos Computational Biology* **2019**, *15*,
[8] C. L. Lawson, R. J. Hanson, *Solving least squares problems* Philadelphia **1995**
[9] R. Gaujoux, C. Seoighe *Infect Genet Evol* **2012**, *12*,913-21.
[10] Y. Zhong, Y. W. Wan, K. Pang, L. M. Chow, Z. Liu *BMC Bioinformatics* **2013**, *14*,89.
[11] B. Rowland, R. Huh, Z. Hou, C. Crowley, J. Wen, Y. Shen, M. Hu, P. Giusti-Rodriguez, P. F. Sullivan, Y. Li *PLoS Genet* **2022**, *18*,e1010102.
